# Projections of meteorological drought severity-duration variations based on CMIP6

**DOI:** 10.1038/s41598-024-55340-x

**Published:** 2024-02-29

**Authors:** Farhad Behzadi, Saman Javadi, Hossein Yousefi, S. Mehdy Hashemy Shahdany, Ali Moridi, Aminreza Neshat, Golmar Golmohammadi, Rahimeh Maghsoudi

**Affiliations:** 1 Department of Water Engineering, Faculty of Agricultural Technology, University of Tehran, Tehran, Iran; 2 Department of Water and Environmental Engineering, Faculty of Civil, Shahid Beheshti University, Tehran, Iran; 3 Department of GIS/RS, Faculty of Natural Resources and Environment, Science and Research Branch, Islamic Azad University, Tehran, Iran; 4 Department of Soil, Water and Ecosystem Sciences, Ranch Cattle REC, University of Florida, Gainesville, USA

**Keywords:** Climate sciences, Environmental sciences, Hydrology, Natural hazards

## Abstract

This research utilized the outputs from three models of the Coupled Model Intercomparison Project Phase 6 (CMIP6), specifically CanESM5, GFDL-ESM4, and IPSL-CM6A-LR. These models were used under the SSP1-2.6 and SSP5-8.5 scenarios, along with the SPI and SPEI, to assess the impacts of climate change on drought in Iran. The results indicated that the average annual precipitation will increase under some scenarios and decrease under others in the near future (2022–2050). In the distant future (2051–2100), the average annual precipitation will increase in all states by 8–115 mm. The average minimum and maximum temperature will increase by up to 4.85 ℃ and 4.9 ℃, respectively in all states except for G2S1. The results suggest that severe droughts are anticipated across Iran, with Cluster 5 expected to experience the longest and most severe drought, lasting 6 years with a severity index of 85 according to the SPI index. Climate change is projected to amplify drought severity, particularly in central and eastern Iran. The SPEI analysis confirms that drought conditions will worsen in the future, with southeastern Iran projected to face the most severe drought lasting 20 years. Climate change is expected to extend drought durations and increase severity, posing significant challenges to water management in Iran.

## Introduction

Significant changes has been proved to occur due to increase in the concentration of the greenhouse gases and climate change^1^. The extreme climate events can cause economic losses and impact people in many ways^2^. Thus, understanding the spatiotemporal characteristics of future droughts in the context of climate change is vitally important in designing the relevant adaptation and mitigation measures. Drought is a common natural disaster that frequently occurs worldwide and cannot be managed and mitigated as promptly as other natural disasters such as floods, wind storms, and even earthquakes^3^. Drought always occurs due to precipitation deficiency and is aggravated by higher temperature and evapotranspiration than normal conditions, leading to reduced availability of water resources^4–7^. Moreover, droughts are among the most widespread climatic extremes, damaging ecosystems and deteriorating the land carbon sink^8,9^.

Global Climate Models (GCMs) have been used as a primary tool for examining the past and future changes in climate extremes to determine the effects of climate change on drought. A new generation of GCMs has been recently developed for the Coupled Model Intercomparison Project Phase 6 (CMIP6) experimental design and organization^10^. A new set of emissions and land-use scenarios was considered in CMIP6 under new features of societal development, namely the shared socioeconomic pathways (SSPs). The output of CMIP6 has been used in various studies to predict drought and meteorological data worldwide. For example, using CMIP6 models and SSP scenarios, Su et al.^11^ and Li et al.^12^ determined drought conditions over China and northwest China, respectively. Combining 20 CMIP6 model outputs indicated that climate change will increase precipitation in the Northern region of Egypt by 37% and by 54% for SSP119 and SSP126, respectively, and decrease by 35% for both scenarios in its southwestern region^13^.

Almazroui et al.^14^ used the CMIP6 models (under the three scenarios of SSP1-2.6, SSP2-4.5, and SSP5-8.5) to determine the impacts of climate change on precipitation and temperature for the periods 2030–2059 and 2070–2099 in Africa. The outputs from CMIP6 models were used. Their results showed that, generally, the average annual temperature would increase by 1.2–4.4 ℃ (max. 5.6 ℃ in the Sahara) and the average annual precipitation by 4.8–15.2% in Africa. Suitable planning and issuing early warnings are necessary to determine how climate change causes droughts in various regions. In the same vein, considering emissions scenarios by 2100, Ukkola et al.^15^ reported that CMIP6 models yielded systematic and coherent patterns. Using SSP scenarios, the outputs from CMPI6 models and the SPEI index, Su et al.^11^ determined future drought characteristics for three periods (2021–2040, 2041–2060, and 2081–2100) in China on a 12-month scale. The results indicated that the CMIP6 models had a suitable capability in monitoring of future droughts. Shrestha et al.^16^ also studied droughts in regions in India during 2015–2044 using CMIP6 models and the self-calibrating Palmer Drought Severity Index (scPDSI).

Supharatid and Nafung^17^ used the output of CMIP6 models under the two SSP2-4.5 and SSP5-8.5 scenarios and the Standardized Precipitation Evapotranspiration Index (SPEI) to investigate the impact of climate change on drought over Southeast Asia. Their results showed that the projected drought characteristics show relatively longer durations, higher peak intensities, and more severities under SSP5-8.5, while the higher number of events are projected under SSP2-4.5. Overall, the SPEI-12 over SEA displays significant regional differences with decreasing dryness trend toward the twenty-first century. Also, Li et al.^18^ assesses the duration, frequency, and intensity of drought events in the Asian drylands based on nine CMIP6 models. The results show that a high percentage of land area is experiencing significant drought intensification of 65.1%, 89.9%, and 99.8% under Shared Socioeconomic Pathways (SSP) 126, SSP245, and SSP585 scenarios, respectively. Furthermore, the future droughts will become less frequent but longer in duration and more intense, with even more severe future droughts predicted for northwest China and western parts of Uzbekistan and Kazakhstan.

Iran, located in an arid and semi-arid region, was selected to study changes in precipitation and temperature (minimum and maximum) utilizing the data at 92 synoptic meteorological stations in the near future (2022–2050) and distant future (2051–2100). Moreover, Delaunay Triangulation Clustering, the standardized precipitation index (SPI) and Standardized Precipitation Evapotranspiration Index (SPEI) were used to study drought in Iran. The bivariate (duration and severity) drought analysis was performed using the copula functions (Clayton, Frank, and Gumbel) to calculate the drought return periods.

## Methods and materials

### Study area

**Figure 1:**
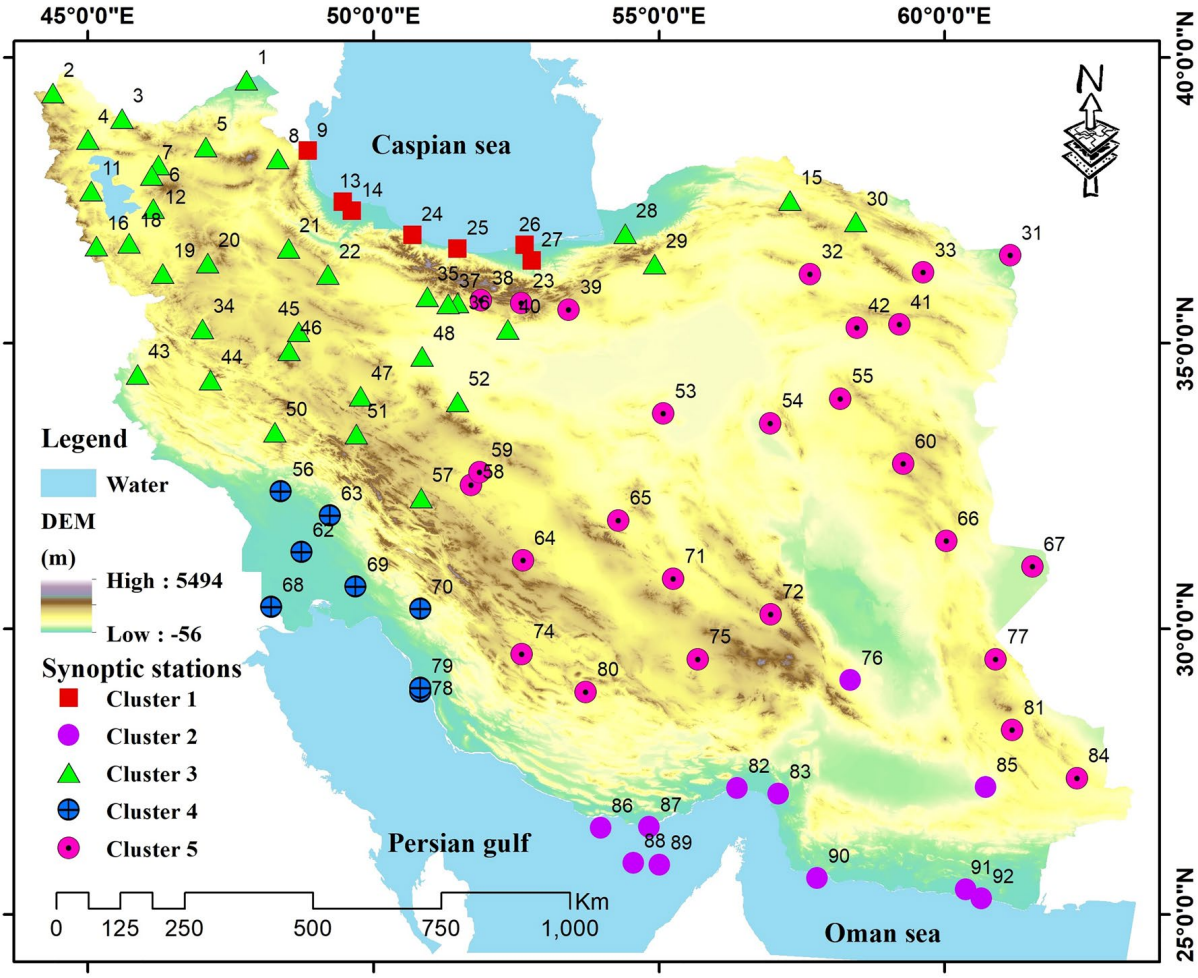
The study area; the study area (Iran) and the synoptic stations used in this research. Moreover, regarding the clustering process carried out in this study, the stations located in the same cluster are marked by the same color. Map created by authors using ESRI ArcGIS Desktop v10.7 (www.esri.com).

Iran is a vast country with varied topography in the Middle East, covering an area of about 154 million hectares between latitudes 23°–41.5° and longitudes 41.5°–67.5°. It is bordered by the Caspian Sea from the north and the Persian Gulf, and the Sea of Oman from the south. Its two main mountain ranges are Alborz in northern Iran, beginning in the Republic of Azerbaijan and extending to eastern Iran and reaching Turkmenistan and Afghanistan and Zagros beginning from the coastal band of Lake Van in southeastern Turkey, crossing several Iranian provinces and reaching southeastern Iran (Fig. 1). These two mountain ranges cause the varied weather conditions throughout Iran. The Zagros Mountain range prevents the advance of rain-producing air masses that mainly enter from the west and cause rains, and its western parts receive more rain than the average annual precipitation in Iran. In addition, the Alborz Mountain range traps the humidity and air currents of the Caspian Sea and prevents them from advancing; hence, its northern parts have more humid climates, but its southern part and also the eastern part of the Zagros Mountain range, which form most of the area in Iran, have arid- and semi-arid climates. The coasts of the Persian Gulf and the Sea of Oman are also influenced and have warm and humid climates. Consequently, Iran can generally be divided into four main climate zones: (1) warm and humid (the southern Iranian coasts), (2) humid temperate (the coasts of the Caspian Sea), (3) cold and mountainous (the slopes of the Zagros and Alborz Mountain ranges) and (4) hot and dry (the central part of Iran).

### Models and scenarios

Three CMIP6 models (CanESM5, GFDL-ESM4, and IPSL-CM6A-LR) were used in this study to determine the impact of climate change on drought in Iran for the period 2022–2100. Table 1 presents the details of the mentioned models. In this research, two SSP scenarios (SSP1 and SSP5) along with the precipitation and minimum and maximum temperatures were used.

**Table 1:** Description of three CMIP6 models and abbreviations for models and scenarios used in the research. Since the results obtained from the models (CanESM5, GFDL-ESM4, and IPSL-CA6A-LR) and the scenarios (SSP1-2.6 and SSP5-8.5) were used in this research, Table 1 presents the abbreviations considered for them to facilitate referring to the results of the models under each scenario. Furthermore, it is noteworthy that descriptions pertaining to the scenarios are provided in SI Table S1, details of the models used in this study are presented in SI Table S2, and the performance of each model in each cluster is depicted in SI Figure S11 to S15 of the Supplementary Information 1.

Model	Institute	Spatial Resolution	Scenarios	State
CanESM5	Canadian Centre for Climate Modelling and Analysis (Canada)	2.8 × 2.8	SSP1-2.6, SSP5-8.5	G1S1, G1S4
GFDL-ESM4	Geophysical Fluid Dynamics Laboratory (United States)	1.0 × 1.3	SSP1-2.6, SSP5-8.5	G2S1, G2S4
IPSL-CM6A-LR	L’Institute Pierre-Simon Laplace (France)	1.3 × 2.5	SSP1-2.6, SSP5-8.5	G3S1, G3S4

G1S1 (CanESM5: SSP1-2.6), G1S4 (CanESM5: SSP5-8.5), G2S1 (GFDL-ESM4: SSP1-2.6), G2S4 (GFDL-ESM4: SSP5-8.5), G3S1 (IPSL-CM6A-LR: SSP1-2.6) and G3S4 (IPSL-CM6A-LR: SSP5-8.5).

The Shared Socioeconomic Pathways (SSPs) are a group of new scenarios developed by the climate change research community to facilitate the integrated analysis of future climate change impacts, vulnerabilities, adaptation, and mitigation. SSP scenarios state how socioeconomic factors will influence the world. They have various aspects such as population, economic growth, education, urbanization, and the rate of technological development. Representative Concentration Pathways (RCPs) are another group of scenarios that consider the volume of emitted greenhouse gases and radiative forcing that may occur in the future but do not purposefully consider any socioeconomic narratives^19^. The present research uses a combination of SSP1 with RCP2.6 and SSP5 with RCP8.5. Table 1 shows the abbreviations used for each state to facilitate the presentation of the results obtained from employing the various scenarios. In addition to the data related to the mentioned models, the historical data on precipitation and the maximum and minimum temperatures from 92 synoptic stations in Iran during the base period (1990–2018) have been used as observation data for downscaling and correcting biases of large- scale data of the models.

### Downscaling

The quantile mapping method was used for correcting bias and downscaling the historical observations data in the models. For this purpose, the cumulative distribution function (CDF) for the predictions and empirical data (historical data) were determined. Finally, when there was a new prediction, the value of the predicted quantile was obtained from the CDF of historical predictions, and the obtained number equivalent to the quantile was then factored in as the corrected data in the CDF of predictions^20–22^. If Y_i_ and Z_i_ are the raw and corrected data, F_Si_ and ${F}_{Oi}^{-1}$ represent CDFs of the raw and observation predictions. Eq. (1) shows the quantile mapping for correcting the predicted variable:

1 $${Z}_{i}={F}_{Oi}^{-1}({F}_{Si}\left({Y}_{i}\right))$$

### Clustering

Delaunay triangulation clustering was used to classify the data obtained from the synoptic stations. In mathematics and computational geometry, a Delaunay triangulation for a set of points “S” is the triangular plate D(S) so that no point in the set S is inside any of the peripheral circles of the D(S) triangles^23^. This triangulation, developed by Delaunay, is widely used in various geographic information systems (GIS)^24^. The pseudo-F statistic was then used to determine the optimal number of clusters. One of the main and best methods for determining the optimal number of clusters is to use the pseudo-F statistic^25^, which is calculated from Eq. (2):

2 $$\frac{\mathrm{Pseudo F}\hspace{0.17em}=\hspace{0.17em}(GSS)/(k-1)}{\left(wss\right)/(N-K)}$$

where N is the number of observations, k is the number of clusters, “GSS” is the between-group sum of squares, and “WSS” is the within-group sum of squares.

### Standardized precipitation Index (SPI)

Various methods are available for monitoring drought. In this research, the SPI method was used which is a powerful index that can be easily used to monitor drought^26^. SPI can be calculated using the long-term precipitation data in a region. To this end, a suitable probability distribution function was first fitted to the long-term precipitation data. In this study, the Pearson type III distribution was selected as the probability function, and the density distribution function was obtained from Eq. (3):^27^

3 $$Pe\left(x\right)=\frac{{\left(x-\frac{\mu -2\sigma }{\gamma }\right)}^{\alpha -1}{\text{exp}}\left[\frac{\left(-x+\frac{\mu -2\sigma }{\gamma }\right)}{\beta }\right]}{{\beta }^{\alpha }\Gamma \left(\alpha \right)}$$

Here, μ, σ, and γ represent the location (average value of the series), the scale, and the shape parameters, respectively. Since this function is not defined for ${\text{x}}<\frac{\upmu -2\upsigma }{\upgamma }$ , the cumulative distribution function was obtained from Eq. (4):^28^

4 $${\text{H}}_{{\rho \, \left( {\text{x}} \right) \, = }} {\text{qp }} + \, \left( {{1 } - {\text{ qp}}} \right){\text{ PE}}_{{3}} \left( {\text{x}} \right)$$

Here, qp is the empirical probability $x<\frac{\mu -2\sigma }{\gamma }$, and PE was obtained from Eq. (5):^27^

5 $${\text{PE}}\left({\text{x}}\right)=\frac{{\text{G}}\left[\mathrm{\alpha },\frac{{\text{x}}-\frac{\upmu -2\upsigma }{\upgamma }}{\upbeta }\right]}{\Gamma \left(\mathrm{\alpha }\right)}$$

Here, G is the incomplete gamma function. After calculating the cumulative probability function, the cumulative probability is transformed to the standard normal variable based on Eq. (6), with an average of zero and a standard deviation of 1^26^:

6 $${\text{SPI}}={\text{z}}=\frac{{{\text{P}}}_{{\text{i}}}-\overline{{\text{P}}}}{\upsigma }$$

where $\overline{p }$ is the average monthly precipitation, σ the standard deviation, and p_i_ is the amount of precipitation at the ith time step. This index can be calculated at any time scale^29^. According to this scale, drought will happen when the SPI at a specific period is constantly ≤ − 1. However, the drought duration ends once its value becomes positive^30^.

### Standardized precipitation evapotranspiration index (SPEI)

Multiple indices exist to assess drought, which also consider the temperature variable. In this study, in addition to the SPI index, the Standardized Precipitation Evapotranspiration Index (SPEI) is also used, as in many other studies to investigate the impact of temperature on drought in Iran^17,31–36^ (Liu et al. 2021). The SPEI drought index is calculated based on the relationship between precipitation and evapotranspiration. Unlike precipitation-based drought indices, such as the Standardized Precipitation Index (SPI), this index takes into account the water balance. To calculate the SPEI index, potential evapotranspiration (PET) is first calculated for the period of interest. Then, to calculate the SPEI index, the difference between precipitation and PET is calculated at the desired time scale. There are also multiple methods for calculating potential evapotranspiration, such as Penman–Monteith, Thornthwaite, Hargreaves, Priestley and Taylor, and Jensen and Haise. In this study, due to the simplicity and applicability of the Thornthwaite method and the availability of the required variables, this method is used to calculate PET. The Thornthwaite equation for calculating PET is shown in Eq. (7):^37^

7 $$\begin{gathered} PET = k\left( \frac{10T}{I} \right)^{a} \times \frac{uN}{{360}} \hfill \\ I = \mathop \sum \limits_{1}^{12} ij \hfill \\ i_{j} = 0.09 \times T^{1.5} \hfill \\ \propto = 0.026 \times I + 0.5 \hfill \\ \end{gathered}$$

where $PET$ is potential evapotranspiration ($mm$), $T$ is mean monthly temperature ($^\circ C)$, $U$ is the number of days per month, $N$ is month average sunshine time (h/d) and the empirical coefficient $k$ is 16.

After calculating PET using Eq. (7), the difference between precipitation and potential evapotranspiration for each month is calculated using Eq. (8), which is indicated by WB (Water Balance):

8 $${WB}_{i}={P}_{i}-{(PET)}_{i}$$

where $i$ represents the precipitation on a certain month, ${P}_{i}$ is the precipitation on a certain month ($mm$) and PET is the calculated potential evapotranspiration on a certain month ($mm$).

The ${WB}_{i}$ time series are aggregated at the required time scales ($k$) as follows based on Eqs. (9–10):

9 $${WB}_{i,j}^{k}=\sum_{I=13-k+j}^{12}{WB}_{i-1,l}+\sum_{l=1}^{j}{WB}_{i,l} if j<k$$

10 $${WB}_{i,j}^{k}=\sum_{l=j-k+1}^{j}{WB}_{i,l} if j\ge k$$

where $j$ is the month and $i$ is the year.

Based on the precipitation and PET, the log-logistic probability distribution is then used to fit the difference between the two ($WB$) and the SPEI value corresponding to each $WB$ value is calculated. By applying the same distribution function parameters established for the observed period (1990–2018) and future period (2022–2100), the dry/wet condition of future SPEI can be assessed based on Eqs. (11–13). When the cumulative probability $p<0.5$, the SPEI is calculated as:

11 $$w=\sqrt{-2{\text{ln}}(p)}$$

12 $$SPEI=w-\frac{{c}_{0}+{c}_{1}w+{c}_{2}{w}^{2}}{1+{d}_{1}w+{d}_{2}{w}^{2}+{d}_{3}{w}^{3}}$$

However, when $$p>0.5$$,

13 $$SPEI=-(w-\frac{{c}_{0}+{c}_{1}w+{c}_{2}{w}^{2}}{1+{d}_{1}w+{d}_{2}{w}^{2}+{d}_{3}{w}^{3}})$$

where the constants ${c}_{0}$, ${c}_{1}$, ${c}_{2}$, ${d}_{1}$, ${d}_{2}$, and ${d}_{3}$ are 2.515517, 0.802853, 0.010328, 1.432788, 0.189269, and 0.001308, respectively.

The $WB$ standardization and SPEI calculation is done for the observed and projection periods independently. And finally, the drought severity obtained from the SPEI index is classified as the SPI index.

### Copulas

A Copula is a function forming a bivariate or multivariable distribution based on two or more univariate marginal distribution functions^38^. If X and Y are assumed to be two dependent random variables such as drought severity and duration with a bivariate distribution function F_xy_ and univariate marginal distribution functions F_x_ and F_Y_, the bivariate copula function (C) is then defined as in Eq. (14):^39^

14 $${F}_{XY}\left({\text{X}},{\text{Y}}\right)={\text{C}}\left({F}_{X}\left(X\right), {F}_{Y}\left(Y\right)\right)$$

Copula functions provide a great deal of flexibility in modeling because the marginal distributions can be selected independently from each other in building a multivariate model. Unlike bivariate distribution functions, there is no need for the marginal distributions to follow a specific distribution^40^. Archimedean and elliptical copulas are widely used in drought analysis. This research used three Archimedean copulas (Frank, Clayton, and Gumbel). Three Archimedean copulas were fitted to drought severity and duration data for bivariate analyses. The equations and their parameters are listed in Eqs. (15–17), Respectively:

15 $$C\left(u,v\right)={\left({u}^{-\theta \, }+{\nu }^{-\theta \, }-1\right)}^{\frac{-1}{\theta}}$$

16 $$C\left(u,v\right)=-\frac{1}{\theta }{\text{log}}\left(1+\frac{\left({e}^{-\theta u}-1\right)\left({e}^{-\theta v}-1\right)}{{e}^{-\theta }-1}\right)$$

17 $$C\left(u,v\right)={e}^{-{\left[{\left(-{\text{log}}\left(u\right)\right)}^{\theta }+{\left(-{\text{log}}\left(v\right)\right)}^{\theta }\right]}^{\frac{1}{\theta }}}$$

The general shape of copula functions in Archimedean copulas is defined in Eq. (18):

18 $$C\left(u, v\right)={\phi }^{-1}\left(\phi \left(u\right),\phi \left(v\right)\right) 0<u, v\le 1$$

where u and v are the marginal functions of drought severity and duration, $\phi$ the generator function for copula, $\phi :I\to \left[0,\infty \right)$ is a continuous convex function ${(\upphi }^{\mathrm{^{\prime}}\mathrm{^{\prime}}}\left({\text{u}}\right)>0)$ and strictly decreasing function (ϕ1 = 0, ϕ*u* <). In all functions, θ is the parameter of the copula function showing the dependence between the variables. Three Archimedean copula functions were fitted to the data on drought duration and severity index for bivariate analyses. The condition for using the copula function is a correlation between drought variables, and it is not suitable to use these functions if the variables are not correlated. Kendall, Pearson, and Spearman correlation coefficients were used to study the correlation between the two drought characteristics (severity and duration). Estimations of the parameters of all three functions are necessary to fit Clayton, Frank, and Gumbel functions to the drought data. The nonparametric function of the maximum likelihood estimation (MLE)^41^, which is obtained from Eq. (19), is used to estimate parameter θ^41^:

19 $${\text{L}}\left(\uptheta \right)=\sum_{{\text{k}}=1}^{{\text{n}}}{\text{log}}\left[{{\text{c}}}_{\uptheta }\left\{{{\text{F}}}_{1}\left({{\text{X}}}_{{\text{ik}}}\right),\dots ,{{\text{F}}}_{{\text{p}}}\left({{\text{X}}}_{{\text{pk}}}\right)\right\}\right]$$

Here, c_θ_ is the copula density function, F the marginal distribution function and ${x}_{1k},{x}_{2k},\dots ,{x}_{pk}(k=1,\dots ,n)$ the dependent random variables. Drought can be studied in a bivariate analysis and based on the characteristics of these variables by using the most suitable copula function that has been fitted to the data on drought severity and duration^9,42–46^.

### Drought return period

As hydrological events, droughts include the severity and duration variables expressing their behavior. In common analyses, the variables of various hydrological events are assumed to be independent, increasing the inaccuracy of statistical estimation for these events^47^. The use of multivariate distributions that take the dependence of the variables is a more correct method. In this regard, using copula functions allows simultaneously calculating the probability of occurrence of droughts and their durations and severity indices. Equation (20) is used to simultaneously calculate the probability of the occurrence of droughts and their durations and severity indices so that the values of both variables exceed the threshold levels^48^.

20 $${P}_{SD}=P\left(d\le D and s\le S\right)={F}_{DS}\left(\infty ,\infty \right)-{F}_{DS}\left(d,\infty \right)-{F}_{DS}\left(\infty ,s\right)+{F}_{DS}\left(d,s\right)=1-{F}_{D}\left(d\right)-{F}_{S}\left(s\right)+C({F}_{D}\left(d\right),{F}_{S}\left(s\right))$$

After calculating the simultaneous probability using Eq. (20), the return period is calculated from Eq. (21):

21 $$\mathrm{TSD }=\mathrm{ T}({\text{S}}\ge s, D\ge d)= \frac{E(L)}{P(S\ge s, D \ge d)} = \frac{E(L)}{1-{F}_{D} \left(d\right)- {F}_{S}\left(s\right)+C({F}_{s}\left(s\right), {F}_{D}\left(d\right))}$$

where T_SD_ is the return period together with drought duration and severity index, L is the interval between the beginning of one drought and the beginning of the following one, which is equal to the sum of the successive drought and wet periods, and E (L) is the average of these intervals.

## Results

### Effects of climate change on precipitation

The results indicated that under near future climate (2022–2050), the average annual precipitation in Iran would exhibit an uptrend in G1S1, G1S4, and G2S1 and a downtrend in G2S4, G3S1, and G3S4. The largest increase in the average annual precipitation in Iran will happen in G1S4 (62 mm), and the largest decline in the average annual precipitation in Iran will occur in G3S1 (9 mm). The highest increase in the annual precipitation (233 mm) will be recorded in Cluster 1 (northern Iran) in G1S4, followed by Cluster 3 in G3S1 (188 mm). In addition, the greatest reduction in the annual precipitation (179 mm) in Cluster 1 (northern Iran) will be in G3S1. In the distant future (2051–2100), the average annual precipitation in Iran will increase for all states reaching its maximum value (115 mm) in G1S4. The largest increase in the annual precipitation (331 mm) will also be observed in Cluster 4 (southwest Iran) in G1S4, and the greatest reduction in annual precipitation (137 mm) will occur in Cluster 3 (northwestern Iran) in G3S4.

**Figure 2:**
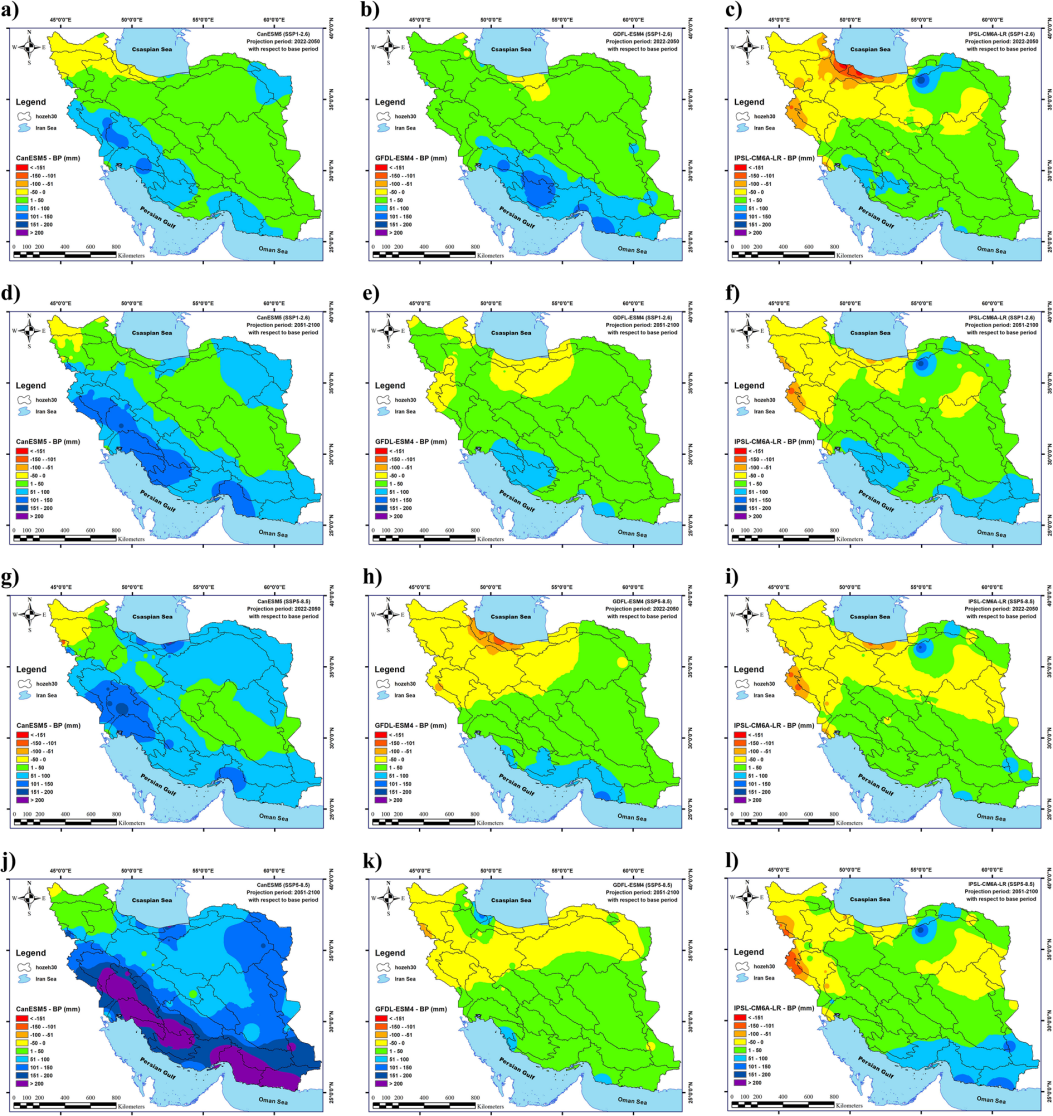
Changes in the long-term average precipitation in Iran under climate change in the near future; changes in the long-term average precipitation in Iran under climate change during 2022–2050 (**a**–**f**) and 2051–2100 (**g**–**l**) determined by the CanESM5, GFDL-ESM4, and IPSL-CM6A-LR models under the SSP1-2.6 and SSP5-8.5 scenarios. (**a**) G1S1, (**b**) G2S1, (**c**) G3S1, (**d**) G1S4, (**e**) G2S4 and (**f**) G3S4 for 2022–2050 and (**g**) G1S1, (**h**) G2S1, (**i**) G3S1, (**j**) G1S4, (**k**) G2S4 and (**l**) G3S4 for 2051–2100. Map created by authors using ESRI ArcGIS Desktop v10.7 (www.esri.com).

The changes in the average, minimum, and maximum precipitation in Iran under climate change conditions in 2021–2050 and 2051–2100 in CanESM5, GFDL-ESM4, and IPSL-CM6A-LR models for two scenarios (SSP1-2.6 and SSP5-8.5) are presented in detail in Table 2 and Fig. 2.

**Table 2:** The effect of climate change on annual precipitation changes.

	Annual precipitation (Future—BP) (mm)	
	(2022–2050)	(2051–2100)
G1S1		
Average	34	57
Maximum	125	159
Minimum	− 39	− 19
G1S4		
Average	62	115
Maximum	233	331
Minimum	− 66	− 35
G2S1		
Average	35	14
Maximum	145	85
Minimum	− 13	− 38
G2S4		
Average	− 4	3
Maximum	132	145
Minimum	− 143	− 79
G3S1		
Average	− 9	8
Maximum	188	171
Minimum	− 179	− 112
G3S4		
Average	− 4	8
Maximum	157	174
Minimum	− 120	− 137

### Effects of climate change on the minimum and maximum temperatures

According to the results, in the near future (2022–2050), the average minimum temperature in Iran will increase in all states except for G2S1. The largest increase in average minimum temperature will be recorded for G1S4 and G3S4 (1.55 ℃ and 1.45 ℃, respectively). The average minimum temperature in Iran will decline in G2S1 by 0.02 ℃. The highest increase in average minimum temperature (2.82 ℃) will be recorded in cluster 5 (the Central Iranian Plateau) in G1S4, followed by cluster 5 in G3S4 (2.56 ℃). The largest decrease in average minimum temperature (0.57 ℃) will be observed in cluster 5 in G2S1. In the distant future (2051–2100), the average minimum temperature in Iran will increase with the highest increase (5.63 ℃ and 4.85 ℃) recorded in G1S4 and G3S4, respectively. The largest increase in average minimum temperature (8 ℃) will happen in cluster 5 (the Central Iranian Plateau) in G1S4 and the greatest decrease (0.65 ℃) will occur in cluster 5 (the Central Iranian Plateau) in G2S1.

Also, the average maximum temperature in Iran will increase for all states except for G2S1 in the near future (2022–2050). The largest increase in the average maximum temperature in Iran will happen in G3S4 and G1S4 (1.37 ℃ and 1.2 ℃, respectively), and the reduction in average maximum temperature (0.4 ℃) will occur in G2S1. The highest increase in the average maximum temperature (4.13 ℃) will be observed in Cluster 4 (southwest Iran) in G3S4, followed by Cluster 4 in G1S4 (3.97 ℃). The greatest decrease in the average maximum temperature (0.92 ℃) will be recorded in Cluster 3 (western Iran) in G2S1, followed by Cluster 3 (northwestern Iran) in G2S4 (with 0.25 ℃). Similarly, in the distant future (2051–2100), the average maximum temperature in Iran will increase in all states except for G2S1. The largest increase in the average maximum temperature in Iran (4.9 ℃) will occur in G3S4, followed by G1S4 (with 4.87 ℃). G2S1 is the only state where the average maximum temperature will decline (by 0.1 ℃). The greatest increase in the average maximum temperature (7.79 ℃) will be observed in Cluster 3 (western Iran) in G3S4, and the largest decrease in the average maximum temperature (0.56 ℃) will also be recorded in Cluster 3 (western Iran) in G2S1.

**Figure 3:**
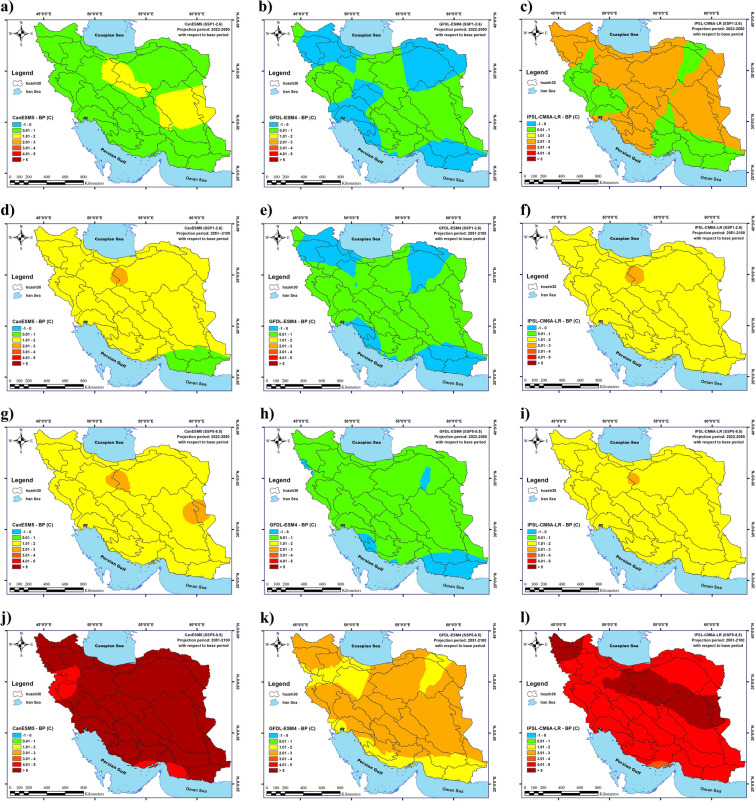
Changes in the long-term average of minimum temperatures in Iran under climate change in the near future; changes in the long-term average of minimum temperatures in Iran under climate change during 2022–2050 (**a**–**f**) and 2051–2100 (**g**–**l**) determined by the CanESM5, GFDL-ESM4, and IPSL-CM6A-LR models under the SSP1-2.6 and SSP5-8.5 scenarios. (**a**) G1S1, (**b**) G2S1, (**c**) G3S1, (**d**) G1S4, (**e**) G2S4 and (**f**) G3S4 for 2022–2050 and (**g**) G1S1, (**h**) G2S1, (**i**) G3S1, (**j**) G1S4, (**k**) G2S4 and (**l**) G3S4 for 2051–2100. Map created by authors using ESRI ArcGIS Desktop v10.7 (www.esri.com).

**Figure 4:**
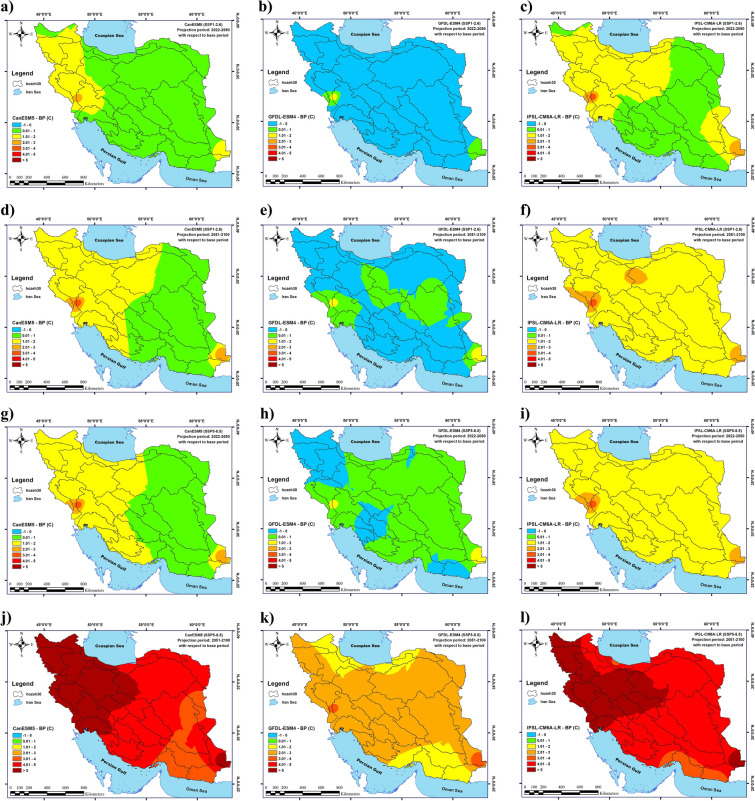
Changes in the long-term average of maximum temperatures in Iran under climate change in the near future; changes in the long-term average of maximum temperatures in Iran under climate change during 2022–2050 (**a**–**f**) and 2051–2100 (**g**–**l**) determined by the CanESM5, GFDL-ESM4, and IPSL-CM6A-LR models under the SSP1-2.6 and SSP5-8.5 scenarios. (**a**) G1S1, (**b**) G2S1, (**c**) G3S1, (**d**) G1S4, (**e**) G2S4 and (**f**) G3S4 for 2022–2050 and (**g**) G1S1, (**h**) G2S1, (**i**) G3S1, (**j**) G1S4, (**k**) G2S4 and (**l**) G3S4 for 2051–2100. Map created by authors using ESRI ArcGIS Desktop v10.7 (www.esri.com).

The changes in the average, minimum, and maximum temperatures in Iran under climate change conditions in 2021–2050 and 2051–2100 in CanESM5, GFDL-ESM4, and IPSL-CM6A-LR models for two scenarios (SSP1-2.6 and SSP5-8.5) are presented in detail in Table 3 and Figs. 3–4.

**Table 3:** The effects of climate change on annual temperature changes.

	Annual temperature (future—BP) (c)			
	Minimum temperature		Maximum Temperature	
	(2022–2050)	(2051–2100)	(2022–2050)	(2051–2100)
G1S1				
Average	0.86	1.44	0.76	1.26
Maximum	1.92	2.67	3.47	4.03
Minimum	0.51	0.88	0.14	0.48
G1S4				
Average	1.55	5.63	1.2	4.87
Maximum	2.82	8	3.97	7.79
Minimum	1.01	4.03	0.5	3.01
G2S1				
Average	− 0.06	0.07	− 0.4	− 0.1
Maximum	0.7	0.78	2.19	2.39
Minimum	− 0.57	− 0.65	− 0.92	− 0.56
G2S4				
Average	0.21	2.12	0.09	2.09
Maximum	1.07	3.12	2.66	4.55
Minimum	− 0.51	1.33	− 0.25	0.87
G3S1				
Average	1.12	1.56	1.13	1.57
Maximum	2.27	2.72	3.74	4.29
Minimum	0.83	1.19	0.63	0.87
G3S4				
Average	1.45	4.85	1.37	4.9
Maximum	2.56	6.51	4.13	7.73
Minimum	1.14	3.69	0.81	2.8

### Bivariate drought analysis using copulas, SPI and SPEI

Delaunay triangulation clustering was used to cluster the synoptic stations and study drought in the clusters. The input vector included 39 members: elevation, longitude, latitude, average monthly precipitation (12 members), average monthly minimum temperature (12 members), and average monthly maximum temperature (12 members). Optimal number of clusters was determined in ArcGIS. Five clusters were selected as the optimal clusters using the pseudo-F statistic. Synoptic stations 24, 83, 46, 69, and 55 were selected as the representatives of Clusters 1–5, respectively. Figure 1 shows the synoptic stations and the clusters.

After ensuring a suitable correlation between the drought variables and determining the number of clusters, three functions (Clayton, Frank, and Gumbel) were used in the bivariate drought analysis. The important steps in using copula functions are selecting an appropriate marginal distribution function for the variables, followed by selecting the superior copula function. If the same marginal distribution is not used for the variables, criteria such as the Akaike information criterion (AIC) or the Bayesian information criterion (BIC) are used. However, since the same marginal function and the same number of estimated parameters were used in this research, the copula function that found the highest maximum likelihood estimation (MLE) was selected as the superior one^49^. Table 4 lists the superior copula functions for each cluster and state.

**Table 4:** The results of selecting the best copula function (based on SPI). Table 4 shows the results of evaluating the copula functions for each cluster in each state (G1S1 to G3S4). The superior copula functions had the highest MLE values.

Models and their scenarios with copulas	Cluster				
	1	2	3	4	5
G1S1					
Clayton	29.21	28.31	38.32	27.35	28.99
Frank	43.18	50.66	32.81	43.65	34.57
Gumbel	41.71	55.27	33.78	44.17	35.37
G1S4					
Clayton	27.62	32.7	28.97	38.86	22.29
Frank	38.91	45.85	38.88	40.94	36.18
Gumbel	37.85	44.1	36.19	35.72	35.48
G2S1					
Clayton	31.90	28.87	31.73	33.83	26.14
Frank	59.03	52.90	36.79	40.85	39.96
Gumbel	65.15	56.88	26.64	40.12	43.19
G2S4					
Clayton	29.28	24.00	35.35	32.60	29.17
Frank	53.23	43.45	39.01	45.10	43.73
Gumbel	50.50	43.45	38.45	46.04	44.07
G3S1					
Clayton	22.55	27.56	28.50	20.67	29.65
Frank	36.13	45.34	45.15	34.03	39.55
Gumbel	38.62	46.15	42.63	34.12	37.28
G3S4					
Clayton	25.62	30.31	16.61	20.60	35.59
Frank	41.00	56.66	30.94	35.34	37.02
Gumbel	42.19	57.70	32.27	37.06	33.37

**Figure 5:**
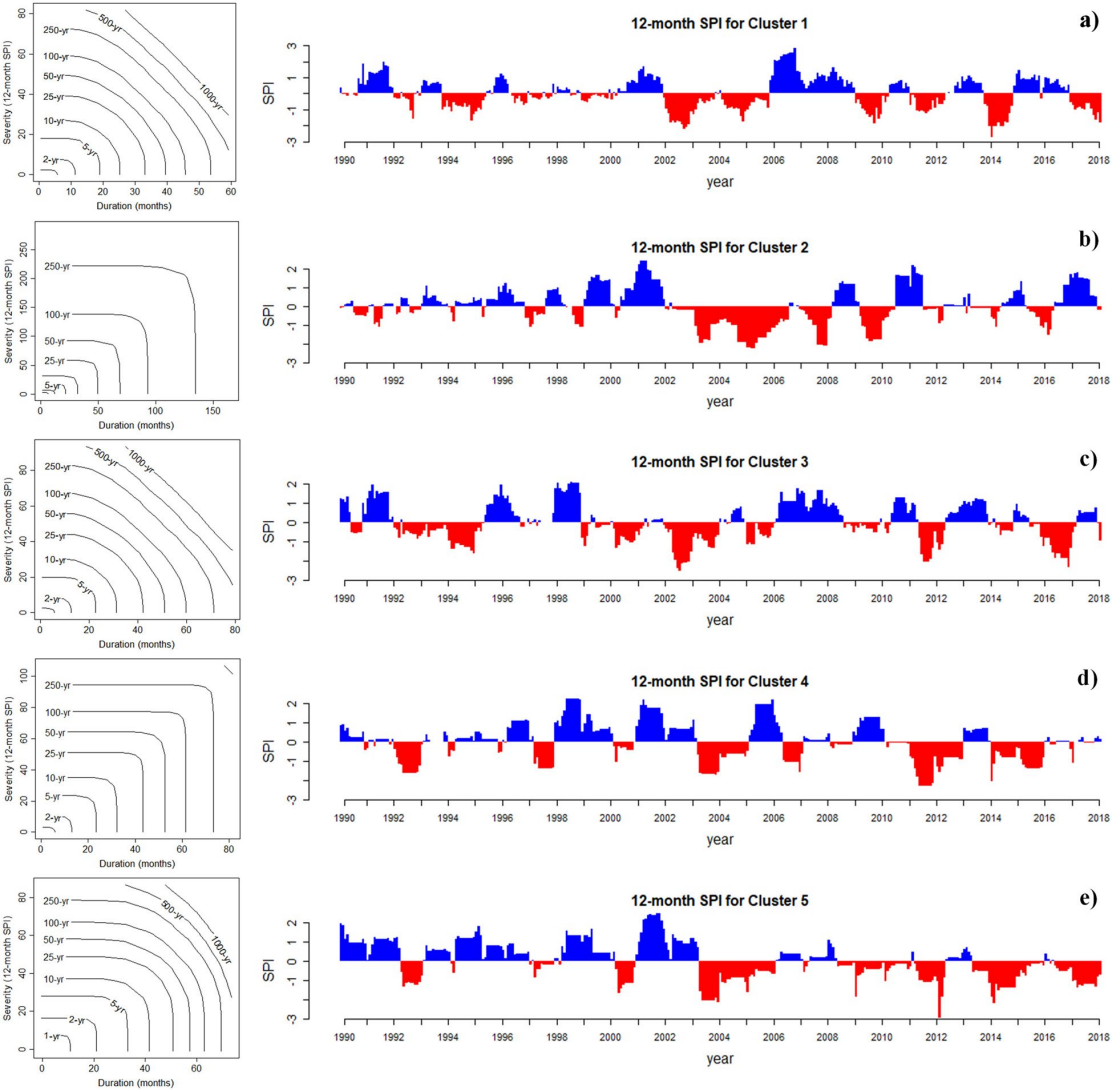
Joint drought returns periods (years) and meteorological drought index (SPI) on a 12-month scale in the observation period (1990–2018) for each of the five clusters in Iran. (**a**) Cluster 1, (**b**) Cluster 2, (**c**) Cluster 3, (**d**) Cluster 4 and (**e**) Cluster 5.

**Figure 6:**
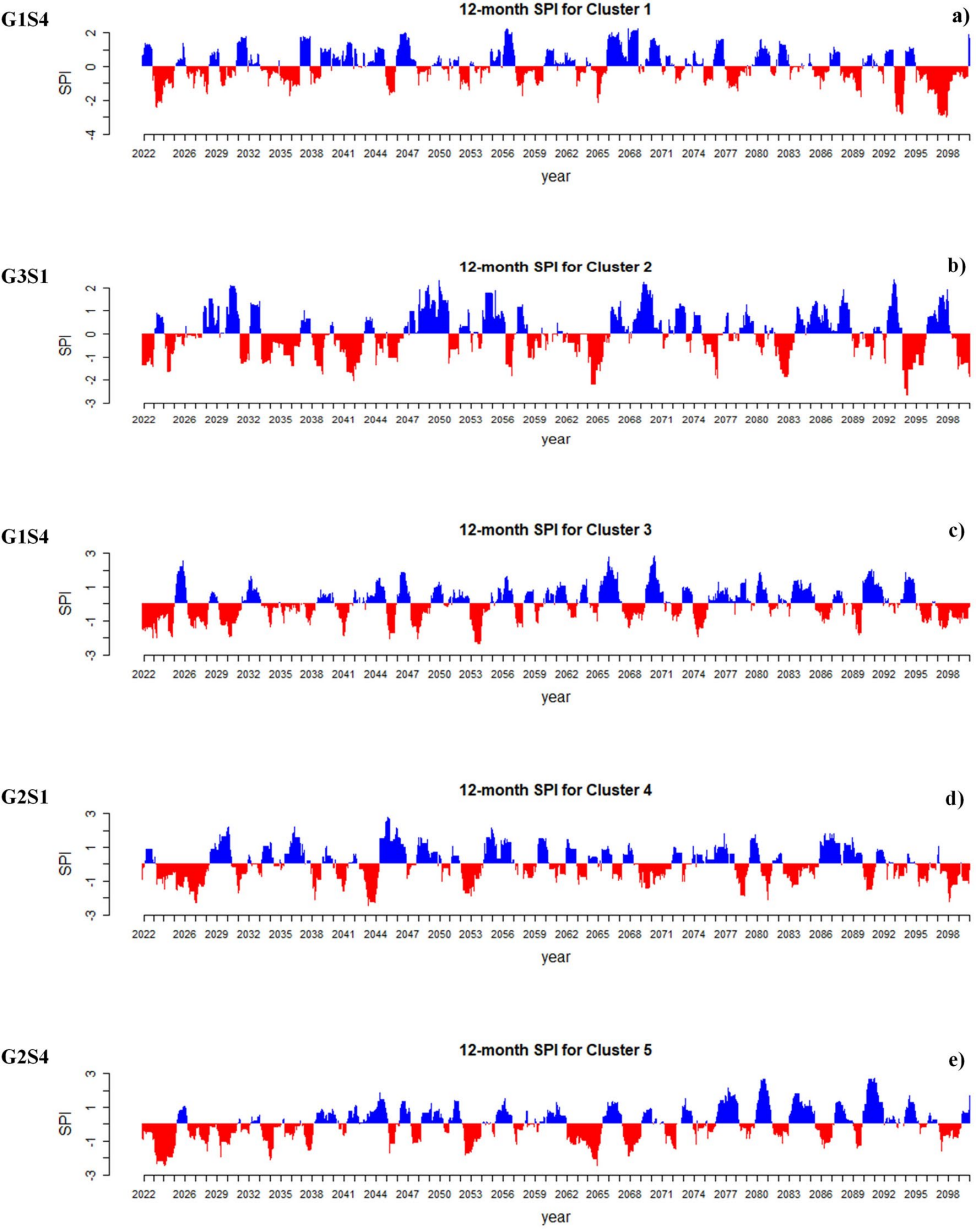
The most severe drought based on meteorological drought index (SPI) on a 12-month scale in the future period (2022–2100) for each of five clusters in Iran. (**a**) Cluster 1, (**b**) Cluster 2, (**c**) Cluster 3, (**d**) Cluster 4 and (**e**) Cluster 5.

**Figure 7:**
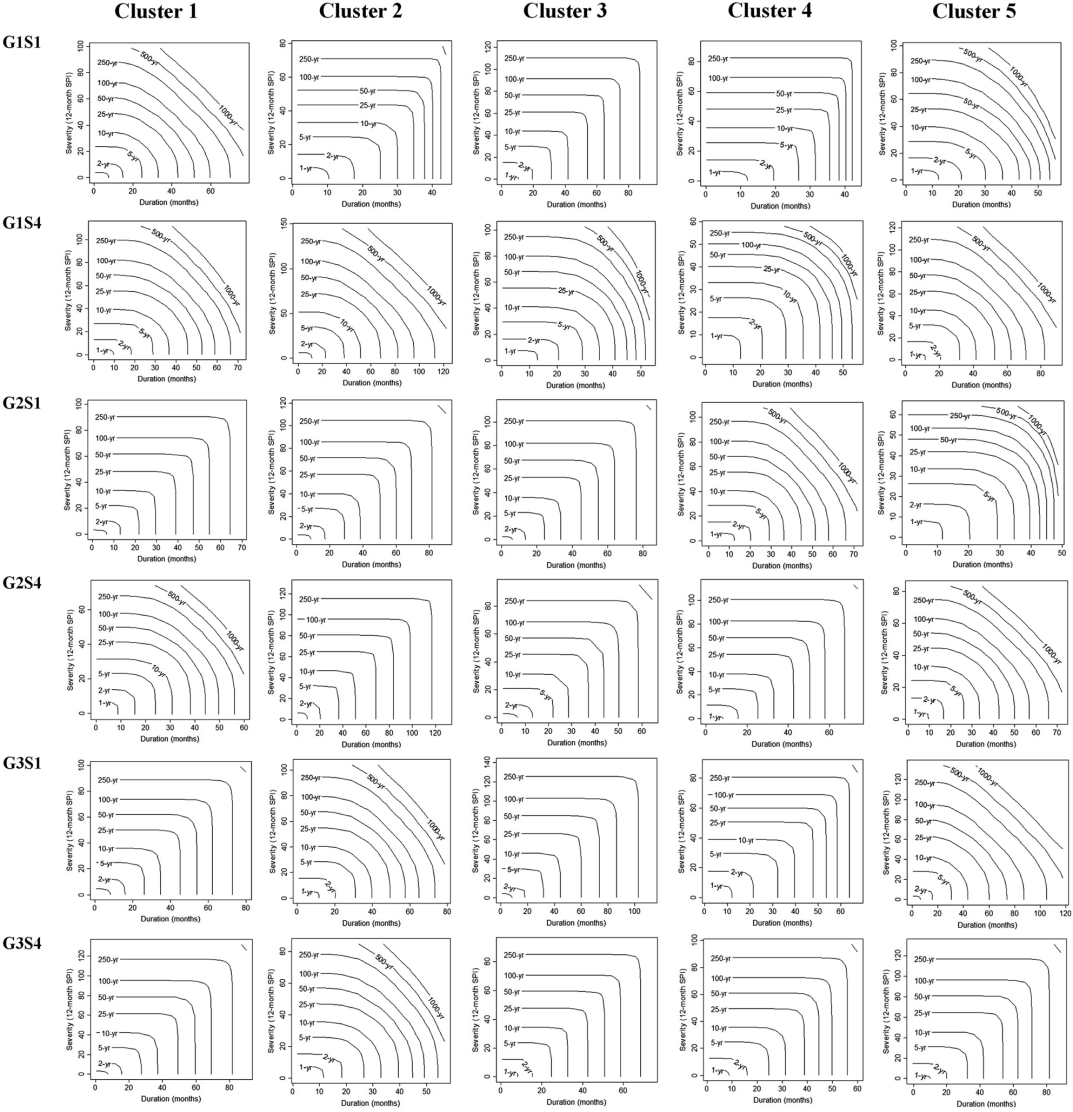
Joint drought returns periods (years) in the future period (2022–2100) for each of the five clusters in Iran under climate change; simultaneous drought severity and duration distribution probability and drought return period under climate change during that determined by the models CanESM5, GFDL-ESM4, and IPSL-CM6-LR and the two scenarios SSP1-2.6 and SSP5-8.5 for each of the five clusters (based on SPI).

Joint drought returns periods (years) and meteorological drought index (SPI) on a 12-month scale in the observation period (1990–2018) for each of the five clusters in Iran presented in Fig. 5. This research shows that the most severe droughts are expected to happen in Iran, considering the SPI index. Based on the results presented in Fig. 6, on average, 51% of the future period (2022–2100) will be under drought conditions in each cluster. Under future climate change, the longest and most severe drought will occur in Cluster 5, lasting for 6 years (2026–2032) with a severity index of 85 (G1S4). The climate change will have its most severe impact on the central and eastern Iran. In contrast, the most severe drought in the base period in this region lasted for 52 months with a severity index of 59. The second most severe drought will occur in Cluster 1 (northern Iran), and this region (considered a high precipitation region in Iran) will face a severe drought lasting for 5 years with a severity index of 73 (G1S4). However, the most severe score in this region in the base period lasted 23 months with a severity index of 26. The most severe drought in Cluster 2 during the base period lasted 3 years with a severity index of 30; however, in the future and under climate change conditions, the most severe drought will last for 5 years with a severity index of 60 (G3S1). Also, in Cluster 3 the most severe drought in the based period lasted for 3 years with a severity index of 38; however, will last for 3 years with a severity index of 59 (G1S4) and in Cluster 4, the most severe drought in the base period continued for 35 months with a severity index of 34, but it will last for 5 years with a severity index of 67 (G2S1) under climate change. It is worth mentioning that only the most severe droughts based on the SPI index are illustrated in Fig. 6, and all results of this index under different states are depicted in SI Figure S1 to S5 of the Supplementary Information 2. Figure 7 present the simultaneous probability distribution of severity-duration and the return period for the base period and each of the clusters and states. As shown, the longest drought return period will increase from 250 years in the base period (1990–2018) in Cluster 2 (southeastern Iran) in G1S4, G3S1, and G3S4, and in Cluster 4 (southwestern Iran) in G1S4 and G2S1 to 1000 years in the future (2022–2100). However, for Cluster 1 (Caspian Sea coasts), Cluster 3 (western and northwestern Iran), and Cluster 5 (central and eastern Iran), the longest drought return period will remain 1000 years. Moreover, the results indicate that climate change causes drought severity and duration to increase throughout Iran in a similar return period. For example, for droughts with a return period of 25 years, drought duration was 32 months with a severity index of 39 in Cluster 1 in the observation period, but for the future period, the drought period will increase by 18 months and reach 50 months, and its severity index will increase by 55% and reach to 60.8 in state G3S4. In Cluster 2, the drought duration was 50 months with a severity index of 54.4 in the observation period, but the drought duration will increase by 17 months and reach 67 months and its severity by 26% and reach 72.5 in the future in G1S4. In Cluster 3, the drought duration was 42 months, with a severity index of 43.2 in the observation period. However, its duration will increase by 18 months to 60 months and its severity index by 59% and 68.8 in the future in G3S1. For Cluster 4, drought duration was 43 months with a severity index of 52 in the base period, but the drought duration will increase by two months to 45 months and its severity index by 10% to 58 in the future in G3S4. In Cluster 5, the drought duration was 52 months with a severity index of 48.7 during the observation period. However, the drought duration will increase by eight months to 60 months and its severity index by 26% to 61.5 in the future in G3S1.

Many researchers have emphasized the importance of temperature on drought conditions. With increasing temperatures, water demand and evaporation and transpiration rates increase, resulting in more severe and longer-lasting droughts. In Iran, which is considered a dry and semi-arid country, the potential evapotranspiration rate in various regions is very high and the temperature variable is of particular importance to the water balance. In this study, in addition to the SPI index, the Standardized Precipitation Evapotranspiration Index (SPEI) was also investigated, which requires precipitation and temperature data to calculate. As mentioned, using the average temperature data and using the Thornthwaite method, the evaporation and transpiration rate for each month was obtained and by subtracting it from the corresponding monthly precipitation and forming the WB time series, the SPEI index in a 12-month scale was obtained for the base period (1990–2018) and the future period (2022–2100). Like the SPI index, the return period of droughts for each cluster and state was calculated using copula functions.

**Figure 8:**
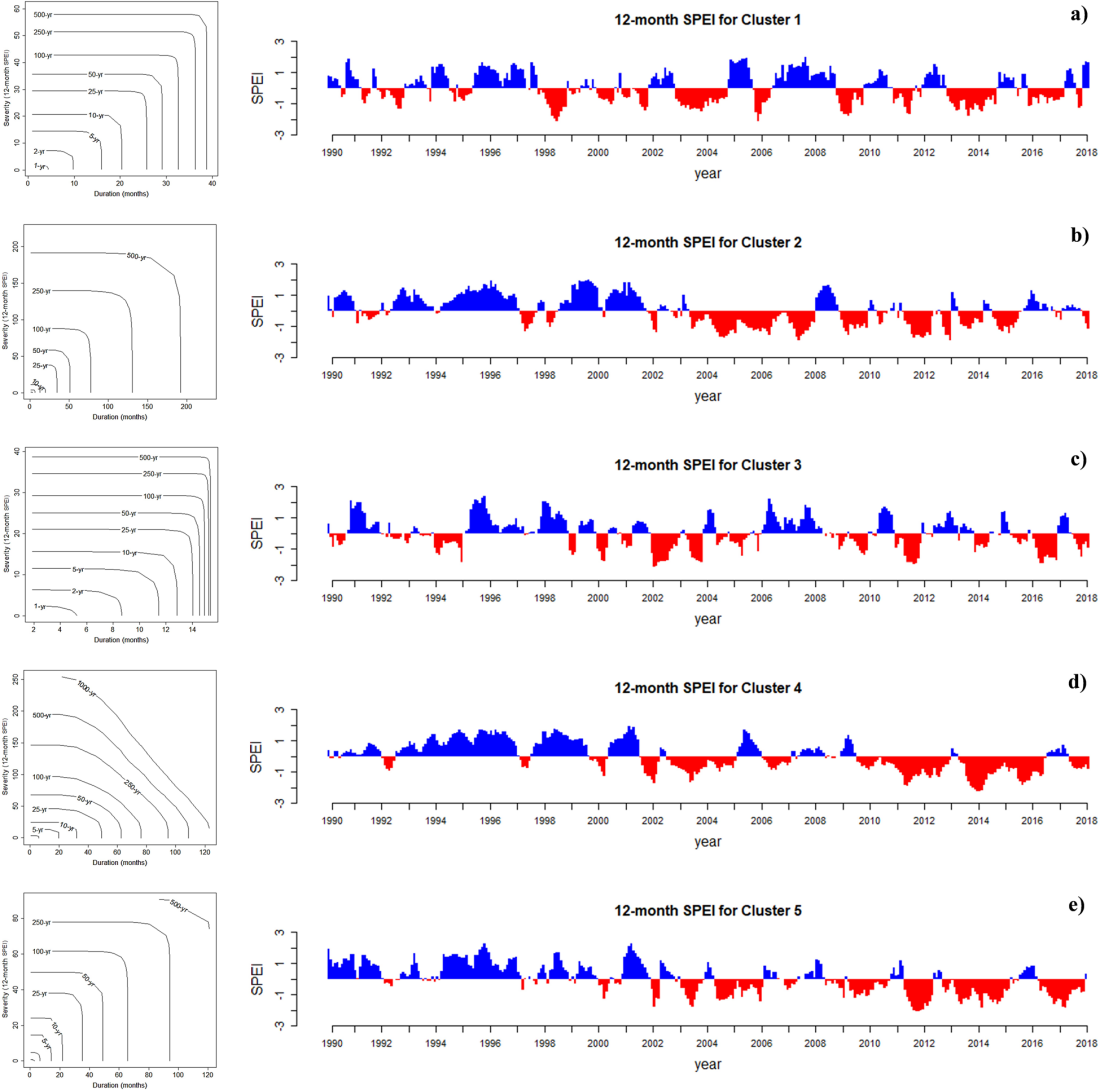
Joint drought returns periods (years) and Standardized Precipitation Evapotranspiration Index (SPEI) on a 12-month scale in the observation period (1990–2018) for each of the five clusters in Iran. (**a**) Cluster 1, (**b**) Cluster 2, (**c**) Cluster 3, (**d**) Cluster 4 and (**e**) Cluster 5.

**Figure 9:**
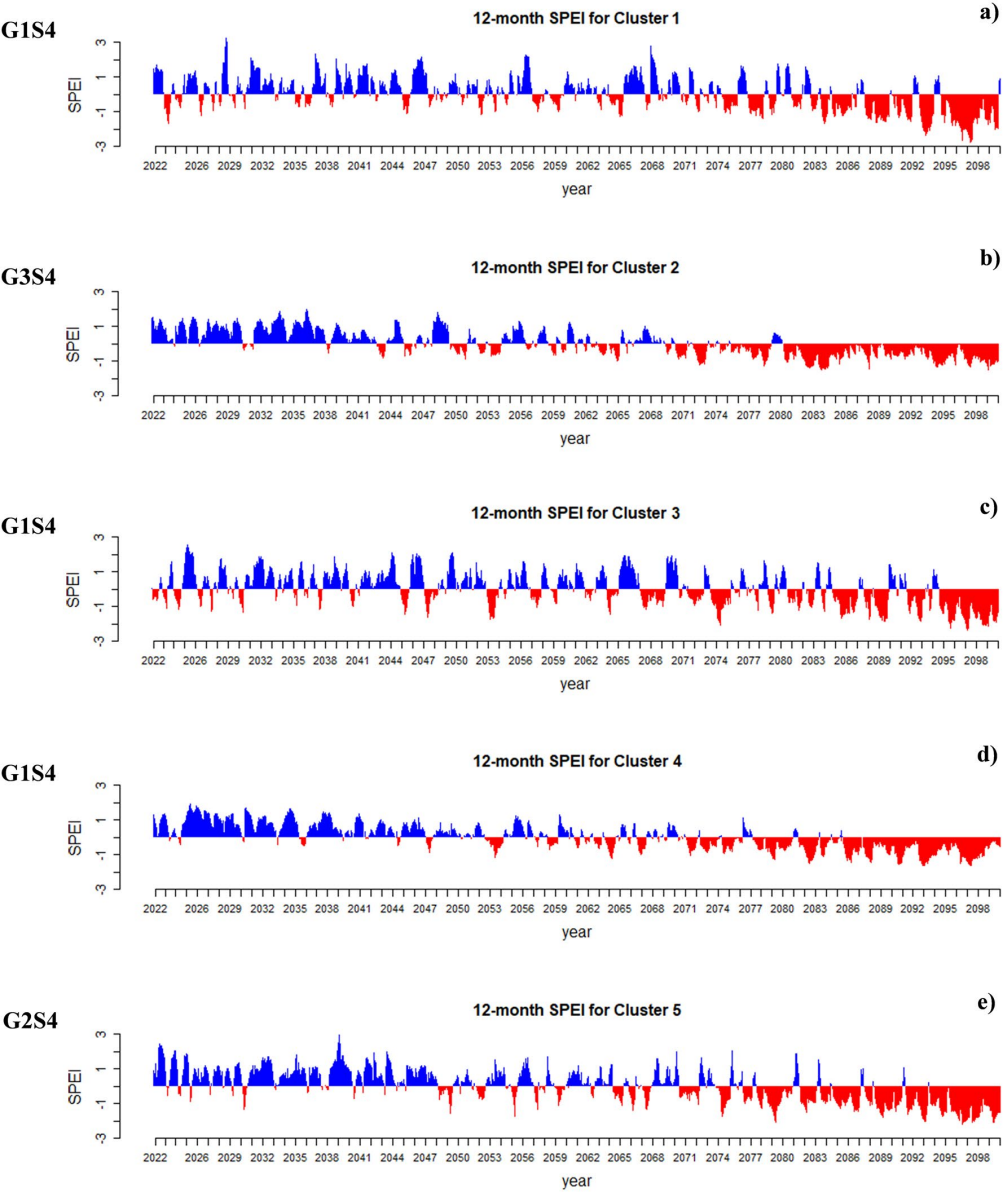
The most severe drought based on Standardized Precipitation Evapotranspiration Index (SPEI) on a 12-month scale in the future period (2022–2100) for each of five clusters in Iran. (**a**) Cluster 1, (**b**) Cluster 2, (**c**) Cluster 3, (**d**) Cluster 4 and (**e**) Cluster 5.

**Figure 10:**
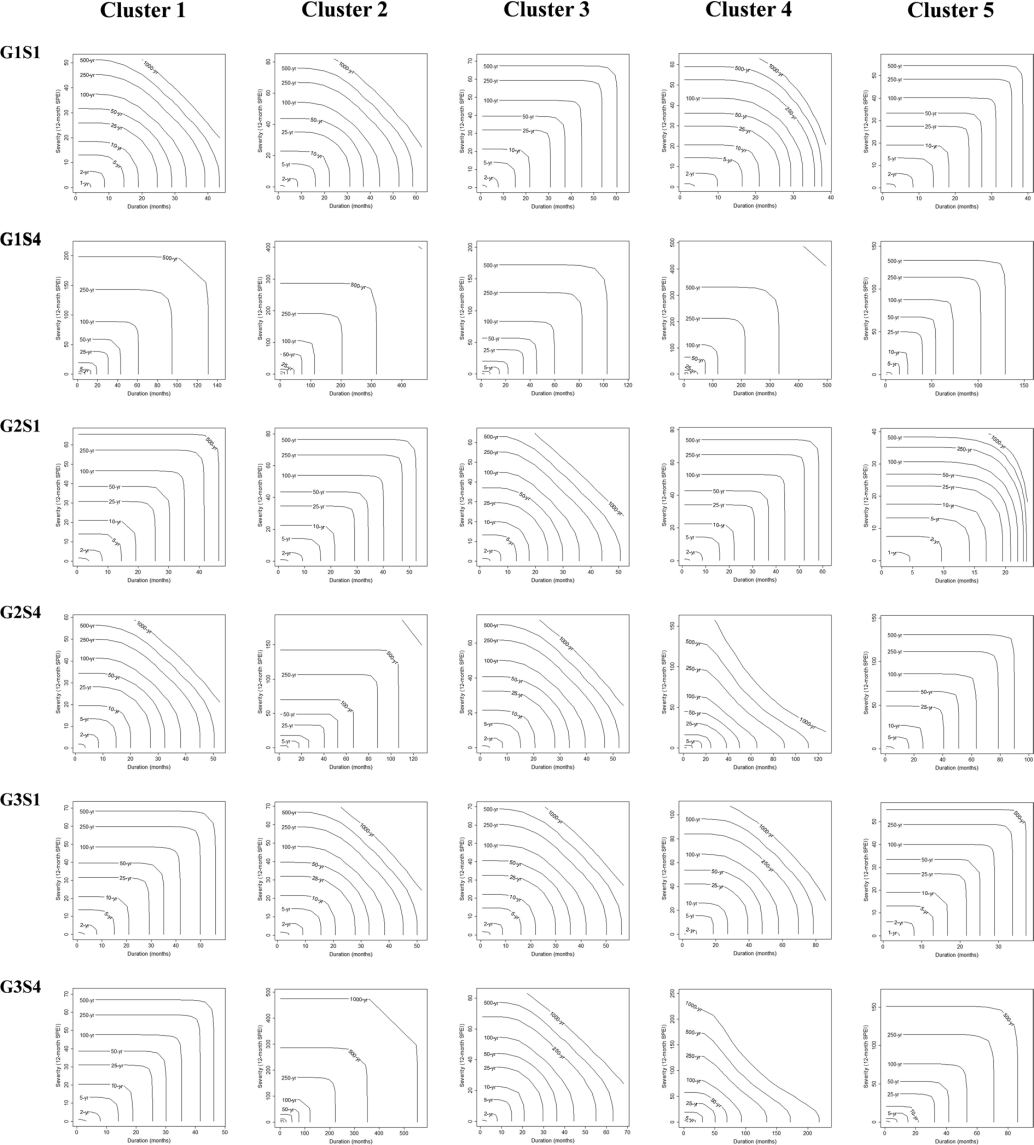
Joint drought returns periods (years) in the future period (2022–2100) for each of the five clusters in Iran under climate change; simultaneous drought severity and duration distribution probability and drought return period under climate change during that determined by the models CanESM5, GFDL-ESM4, and IPSL-CM6-LR and the two scenarios SSP1-2.6 and SSP5-8.5 for each of the five clusters (based on SPEI).

Joint drought returns periods (years) and Standardized Precipitation Evapotranspiration Index (SPEI) on a 12-month scale in the observation period (1990–2018) for each of the five clusters in Iran presented in Fig. 8. In comparison to the observational period, the most severe droughts expected in the future for each of the clusters under each of the states are shown in Fig. 9. It is observed that, similar to the results of the SPI index (Fig. 6), the most severe drought expected in clusters 1 and 3 will occur in state G1S4, and the most severe drought expected for cluster 5 will occur in state G2S4. In general, it is shown in Figs. 3 and 4 that the mean minimum and maximum temperatures in Iran will increase under states G1S4, G2S4, and G3S4 in the period 2051–2100. This will lead to an increase in potential evapotranspiration in Iran. Therefore, with the increase in potential evapotranspiration, the severity of SPEI drought will also increase, which is well shown in Fig. 9. The most severe droughts expected in different regions of Iran have occurred in states G1S4, G2S4, and G3S4. By comparing Figs. 8 and 9, it is clear that the most severe drought expected will occur in cluster 2, which will last for 20 years (2080–2100) with a severity index of 183 (G3S4). Based on this, southeastern Iran will face the most severe drought. This is while the longest drought that occurred in the base period lasted for 56 months and its severity was 55. After that, the most severe drought expected will occur in cluster 4, which will last for 15 years (2085–2100) and with a severity index of 155 (G1S4). The longest drought that occurred in this cluster in the base period lasted for 39 months and its severity was 48. The most severe drought expected in the future for cluster 1 will last for 5 years (2094–2099) with a severity index of 99. The longest drought that occurred in this cluster in the base period lasted for 2 years and its severity was 24. In cluster 3, the most severe drought expected will occur under state G1S4, which will last for 5 years (2095–2100) with a severity of 101. The longest drought that occurred in the base period lasted for 12 months and its severity was 16. Finally, the most severe drought expected for cluster 5 will occur under state G2S4, which will last for 6 years (2094–2098) with a severity index of 118. The longest drought that occurred for this cluster in the base period lasted for 30 months and its severity was 31. It should be noted that Fig. 9 displays only the most severe droughts as indicated by the SPEI index, while all outcomes of this index across various states are demonstrated in SI Figure S6 to S10 of the Supplementary Information 2. Figure 10 present the simultaneous probability distribution of severity-duration and the return period for the base period and each of the clusters and states (based on SPEI). As shown, the longest drought return period will increase from 500 years in the base period (1990–2018) in Cluster 1 (Caspian Sea coasts) in G1S1 and G2S4, and in Cluster 2 (southeastern Iran) in G1S1, G1S4, G2S4, G3S1, and G3S4, and in Cluster 3 (western and northwestern Iran) in G2S1, G2S4, G3S1, and G3S4, and in Cluster 5 (central and eastern Iran) in G2S1 to 1000 years in the future (2022–2100). However, for Cluster 4 (southwestern Iran), the longest drought return period will remain 1000 years. Moreover, the results indicate that climate change causes drought severity and duration to increase throughout Iran in a similar return period. For example, for droughts with a return period of 25 years drought duration was 26 months with a severity index of 29 in Cluster 1 in the observation period, but for the future period, the drought period will increase by 5 months and reach 31 months, and its severity index will increase by 34% and reach to 39 in state G1S4. In Cluster 2, the drought duration was 34 months with a severity index of 39 in the observation period, but the drought duration will increase by 6 months and reach 40 months and its severity by 3% and reach 40 in the future in G2S4. In Cluster 3, the drought duration was 14 months, with a severity index of 21 in the observation period. However, its duration will increase by 16–30 months and its severity index by 67% and 35 in the future in G3S4. For Cluster 4, drought duration was 49 months with a severity index of 46 in the base period, but the drought duration will increase by two months to 51 months but its severity index decreases by 19% to 38 in the future in G3S4. In Cluster 5, the drought duration was 35 months with a severity index of 38 during the observation period. However, the drought duration will increase by 5–40 months and its severity index by 32% to 50 in the future in G1S4. Also, Table 5 lists the superior copula functions for each cluster and state (based on SPEI).

**Table 5:** The results of selecting the best copula function (based on SPEI). Table 5 shows the results of evaluating the copula functions for each cluster in each state (G1S1 to G3S4). The superior copula functions had the highest MLE values.

Models and their scenarios with copulas	Cluster				
	1	2	3	4	5
G1S1					
Clayton	46.12	44.47	41.43	38.86	49.2
Frank	76.68	66.64	62.91	56.4	76.06
Gumbel	73	63.1	67.98	52.51	74.56
G1S4					
Clayton	47.06	32.18	46.77	28.64	34.07
Frank	79.34	50.19	69.69	46.06	53.56
Gumbel	84.35	58.1	70.94	55.75	59.52
G2S1					
Clayton	35.33	44.84	51.72	46.48	46.52
Frank	68.21	63.98	81.91	72.3	66.83
Gumbel	73.78	69.9	78.51	72.5	64.37
G2S4					
Clayton	45.84	41.33	43.89	46.53	26.02
Frank	67.82	55.63	71.94	67.31	42.33
Gumbel	67.09	60.28	71.47	66.99	49.97
G3S1					
Clayton	31.9	47.55	52.73	43.38	50.97
Frank	55.24	71.11	86.55	62.18	80.31
Gumbel	62.82	63.05	80.45	58.6	86.61
G3S4					
Clayton	34.87	33.93	44.32	34.32	37.76
Frank	58.85	40.61	65.58	47.79	59.86
Gumbel	62.38	44.21	62.51	46.54	66

## Discussion

This research intended to answer the question: what impact will climate change have on Iranian meteorological drought? For this purpose, as in the studies by Su et al.^11^, Li et al.^12^, Nashwan and Shahid^13^, and Almazroui et al.^14^, the outputs from three CMIP6 models (CanESM5, GFDL-ESM4, and IPSL-CM6A-LR) and two scenarios from the Sixth Global Climate Change Assessment Report (SSP1-2.6 and SSP5-8.5) were used to determine the impacts of climate change on Iranian weather and evaluate droughts for the near future (2022–2050) and the distant future (2051–2100). The results demonstrated that in the near future (2022–2050), the average annual precipitation will increase in G1S1, G3S1, and G3S4, and the most significant increase (62 mm) will be observed in G1S4. The average annual precipitation will decrease in G2S4, G3S1, and G3S4, and the greatest decrease (9 mm) will be recorded in G3S1. The average minimum and maximum temperatures will increase in all states except for G2S1, and the highest increases in average minimum and maximum temperatures (1.55 ℃ and 1.37 ℃) will be recorded in G1S4 and G3S4, respectively. In the distant future (2051–2100), the average annual precipitation will increase in all states, and the maximum increase (115 mm) will be recorded in G1S4. The average minimum temperature will increase in all states, and the largest increase (5.63 ℃) will be observed in G1S4. The average maximum temperature will increase in all states except for G2S1, and the largest increase (4.9 ℃) will be observed in G3S4. In G2S1, the average maximum temperature will decline by 0.1 °C.

In addition, as in the studies by Su et al.^11^, Li et al.^12^, Ukkola et al.^15^, Shrestha et al.^16^, Li et al.^18^, and Supharatid and Nafung^17^ the outputs from CMIP6 models were used to analyze droughts in the distant future in Iran. This study used the Standardized Precipitation Index (SPI) and Standardized Precipitation Evapotranspiration index (SPEI) on a 12-month scale to monitor drought. However, contrary to the mentioned studies, Delaunay Triangulation Clustering and analysis of drought severity and duration variables employing the copula functions Clayton, Frank, and Gumbel were used to calculate the drought return period for each state and each cluster. According to the various scenarios and models, based on SPI index, 47–51% of the land area in Iran will face drought conditions. The longest drought period will be 6 years and will happen in Cluster 2 from 2022 to 2032. The highest drought severity index (85) during these 6 years will occur in G1S4. The results show that, in identical return periods, droughts with greater severity and longer duration will happen in Iran. Also, based on SPEI index, Droughts across Iran are projected to intensify, becoming more severe and enduring than currently experienced. Southeastern Iran (cluster 2) will bear the brunt of this change, facing a devastating 20-year (2080–2100) drought with a severity index of 183 under state G3S4. Compared to the 1990–2018 baseline, all regions will witness an increase in both drought severity and duration. Furthermore, under various future climate scenarios, the longest drought return period for most clusters is expected to jump from 500 to 1000 years, underlining the dire situation. That is why, in general, climate change will have adverse effects on Iran, and this country will face more difficult and drier conditions. Su et al.^11^ showed that more severe and longer droughts will occur in China. Consequently, suitable management decisions must be adopted beforehand to prevent the destructive effects of climate change in Iran in the distant future and reduce and adapt to the damages inflicted on agriculture, the environment, and the socioeconomic aspects as much as possible. Furthermore, the results of Li et al.^18^ show that, based on the SPEI index, more severe but shorter droughts are projected to occur in northwest China and western parts of Uzbekistan and Kazakhstan. Additionally, the results of Supharatid and Nafung^17^ show that the projected drought characteristics show relatively longer durations, higher peak intensities, and more severities under SSP5-8.5, while the higher number of events are projected under SSP2-4.5. Overall, the SPEI-12 over SEA displays significant regional differences with decreasing dryness trend toward the twenty-first century. In line with this, the results of the present study showed that the results of all three models under the SSP5-8.5 scenario show that the average minimum and maximum temperatures in Iran will increase during 2051–2100. This increase is well reflected in the results of the SPEI index. With the increase in temperature during this period, the potential evapotranspiration rate in Iran will increase, and it is expected that droughts with higher intensity and duration will occur. As shown in the results of Fig. 9, severe droughts will occur in Iran during the period 2080–2100. This study investigates the projection of drought conditions across Iran (located in the Middle East) in terms of duration and intensity. While this research primarily focuses on climate change impact analysis, it is crucial to consider various sources of uncertainty. Although CMIP6 provides finer resolution compared to CMIP5 projections, certain inevitable factors still contribute to uncertainties. These factors include These factors include population growth rate, inequality, international trade, globalization, consumption patterns, environmental policies, development transfers, energy technology changes, fossil fuel constraints, land use alterations, and agriculture^50^. Consequently, there are numerous uncertainties to address in future regional projections. Calculating these uncertainties could warrant a separate research effort, akin to the studies conducted by Athanasiou et al.^51^ and Shiogama et al.^52^.

### Supplementary Information

Supplementary Information 1.

Supplementary Information 2.

## Data Availability

The datasets generated and/or analyzed during the current study are available in the ESGF repository, https://esgf-node.llnl.gov/search/cmip6/.
