# Supplementary material for: Projections of meteorological drought severity-duration variations based on CMIP6
Source: Sci Rep. 2024 Feb 29;14:5027. doi: 10.1038/s41598-024-55340-x (PMC11322161; doi:10.1038/s41598-024-55340-x)
Supplement: Supplementary file 2 — Supplementary Information 2. [file 41598_2024_55340_MOESM2_ESM.docx]

**Supplementary Information for**

**“Projections of Meteorological Drought Severity-Duration Variations based on CMIP6”**

Farhad Behzadi, Saman Javadi, Hossein Yousefi, S. Mehdy Hashemy Shahdany, Ali Moridi, Aminreza Neshat, Golmar Golmohammadi

**Table of Contents**

1. Additional Supplementary Tables
2. Additional Supplementary Figures

**Supplementary Table 1** Summary of SSP narrative (Riahi *et al.*, 2017)

| SSP1 | Sustainability – Taking the Green Road (Low challenges to mitigation and adaptation)  The world shifts gradually, but pervasively, toward a more sustainable path, emphasizing more inclusive development that respects perceived environmental boundaries. Management of the global commons slowly improves, educational and health investments accelerate the demographic transition, and the emphasis on economic growth shifts toward a broader emphasis on human well-being. Driven by an increasing commitment to achieving development goals, inequality is reduced both across and within countries. Consumption is oriented toward low material growth and lower resource and energy intensity. |
| --- | --- |
| SSP2 | Middle of the Road (Medium challenges to mitigation and adaptation)  The world follows a path in which social, economic, and technological trends do not shift markedly from historical patterns. Development and income growth proceeds unevenly, with some countries making relatively good progress while others fall short of expectations. Global and national institutions work toward but make slow progress in achieving sustainable development goals. Environmental systems experience degradation, although there are some improvements and overall the intensity of resource and energy use declines. Global population growth is moderate and levels off in the second half of the century. Income inequality persists or improves only slowly and challenges to reducing vulnerability to societal and environmental changes remain. |
| SSP3 | Regional Rivalry – A Rocky Road (High challenges to mitigation and adaptation)  A resurgent nationalism, concerns about competitiveness and security, and regional conflicts push countries to increasingly focus on domestic or, at most, regional issues. Policies shift over time to become increasingly oriented toward national and regional security issues. Countries focus on achieving energy and food security goals within their own regions at the expense of broader-based development. Investments in education and technological development decline. Economic development is slow, consumption is material-intensive, and inequalities persist or worsen over time. Population growth is low in industrialized and high in developing countries. A low international priority for addressing environmental concerns leads to strong environmental degradation in some regions. |
| SSP4 | Inequality – A Road Divided (Low challenges to mitigation, high challenges to adaptation)  Highly unequal investments in human capital, combined with increasing disparities in economic opportunity and political power, lead to increasing inequalities and stratification both across and within countries. Over time, a gap widens between an internationally-connected society that contributes to knowledge- and capital-intensive sectors of the global economy, and a fragmented collection of lower-income, poorly educated societies that work in a labor intensive, low-tech economy. Social cohesion degrades and conflict and unrest become increasingly common. Technology development is high in the high-tech economy and sectors. The globally connected energy sector diversifies, with investments in both carbon-intensive fuels like coal and unconventional oil, but also low-carbon energy sources. Environmental policies focus on local issues around middle and high income areas. |
| SSP5 | Fossil-fueled Development – Taking the Highway (High challenges to mitigation, low challenges to adaptation)  This world places increasing faith in competitive markets, innovation and participatory societies to produce rapid technological progress and development of human capital as the path to sustainable development. Global markets are increasingly integrated. There are also strong investments in health, education, and institutions to enhance human and social capital. At the same time, the push for economic and social development is coupled with the exploitation of abundant fossil fuel resources and the adoption of resource and energy intensive lifestyles around the world. All these factors lead to rapid growth of the global economy, while global population peaks and declines in the 21st century. Local environmental problems like air pollution are successfully managed. There is faith in the ability to effectively manage social and ecological systems, including by geo-engineering if necessary. |

**Supplementary Table 2 Specifications of CMIP6 models used in this research**

| Institute | Country | Model | No |
| --- | --- | --- | --- |
| Commonwealth Scientific and Industrial Research Organization | Australia | ACCESS-CM2 | 1 |
| Beijing Climate Center Ocean–atmosphere Coupled Model | China | BCC-CSM2-MR | 2 |
| Canadian Earth System Model | Canada | CanESM5 | 3 |
| The Whole Atmosphere Community Climate Model with thermosphere and ionosphere extension | USA | CESM2-WACCM | 4 |
| Flexible Global Ocean-Atmosphere-Land System | China | FGOALS-f3-L | 5 |
| National Oceanic and Atmospheric Administration, Geophysical Fluid Dynamics Laboratory | USA | GFDL-ESM4 | 6 |
| Hadley Centre Global Environment Model in the Global Coupled configuration (UK) | UK | HadGEM3-GC31-LL | 7 |
| Institute for Numerical Mathematics | Russia | INM-CM5-0 | 8 |
| Institute Pierre-Simon Laplace | France | IPSL-CMM6A-LR | 9 |
| Korea Institute of Ocean Science and Technology | South Korea | KIOST-ESM | 10 |
| Atmosphere-ocean coupled model | Japan | MIROC6 | 11 |
| Norwegian Climate Prediction Model | Norway | NorCPM1 | 12 |

**
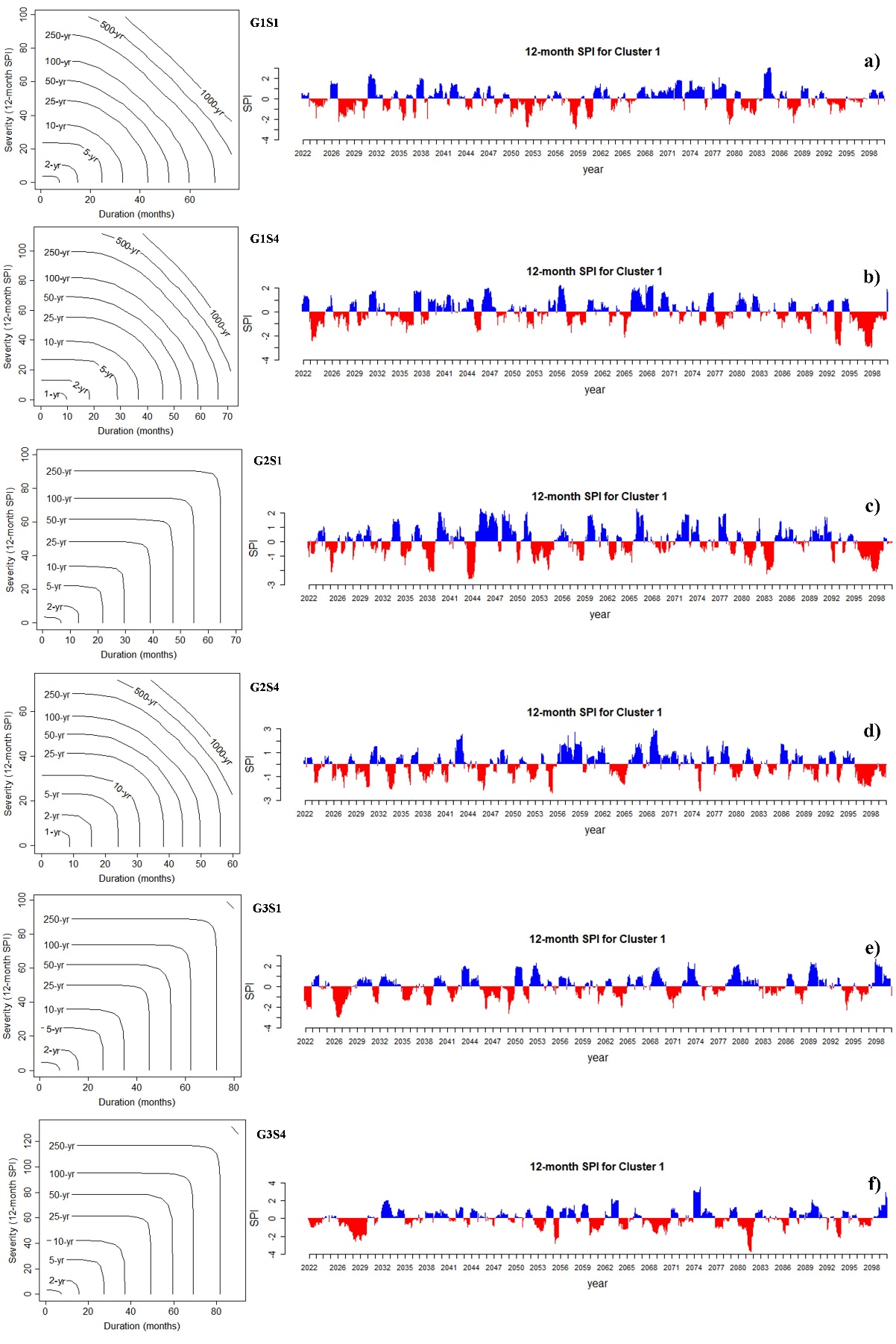
**

**Supplementary Figure 1** Joint drought returns periods (years) and meteorological drought index (SPI) on a 12-month scale in the future period (2022-2100) for cluster 1 in Iran. a) G1S1, b) G1S4, c) G2S1, d) G2S4, e) G3S1 and f) G3S4.


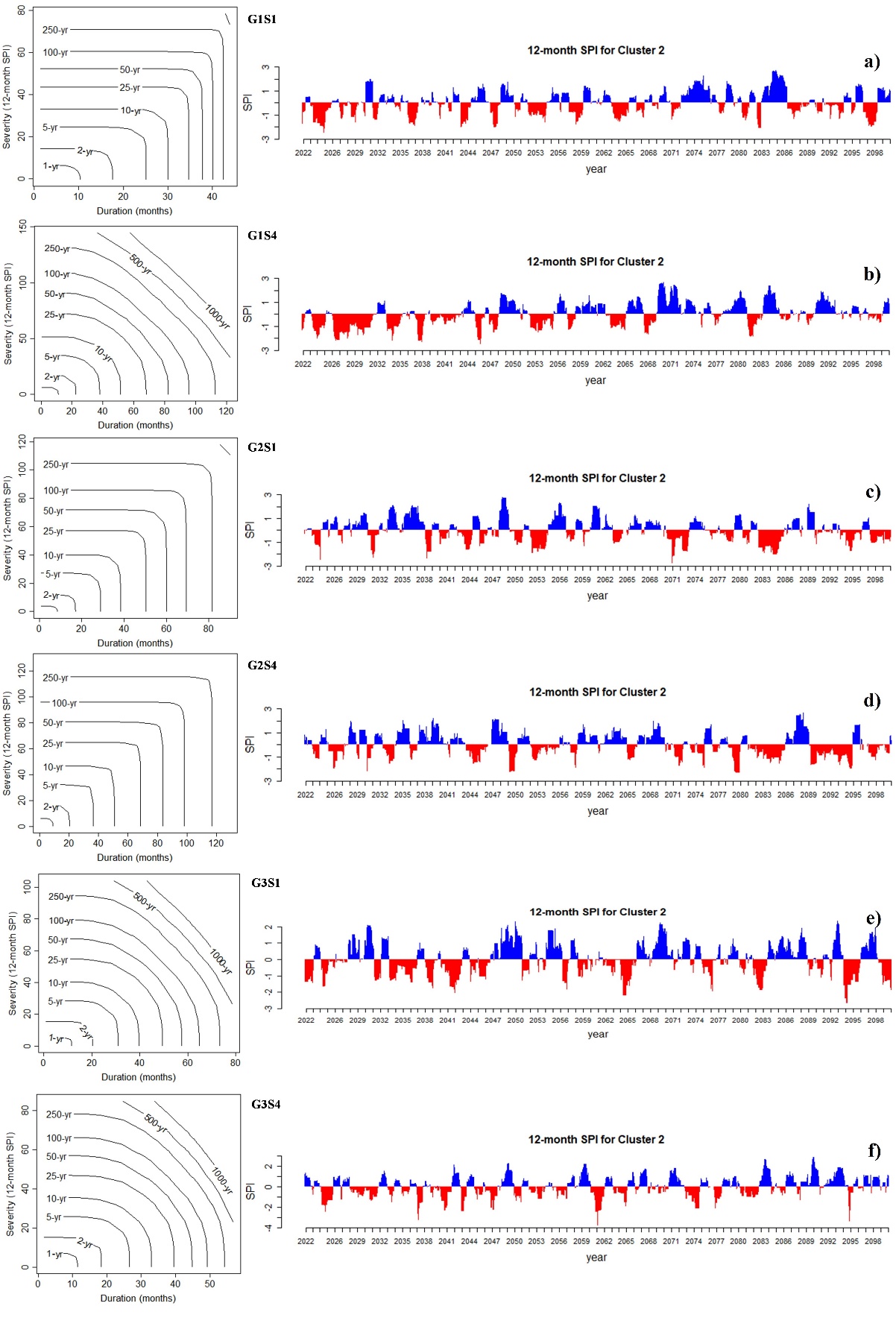


**Supplementary Figure 2** Joint drought returns periods (years) and meteorological drought index (SPI) on a 12-month scale in the future period (2022-2100) for cluster 2 in Iran. a) G1S1, b) G1S4, c) G2S1, d) G2S4, e) G3S1 and f) G3S4.


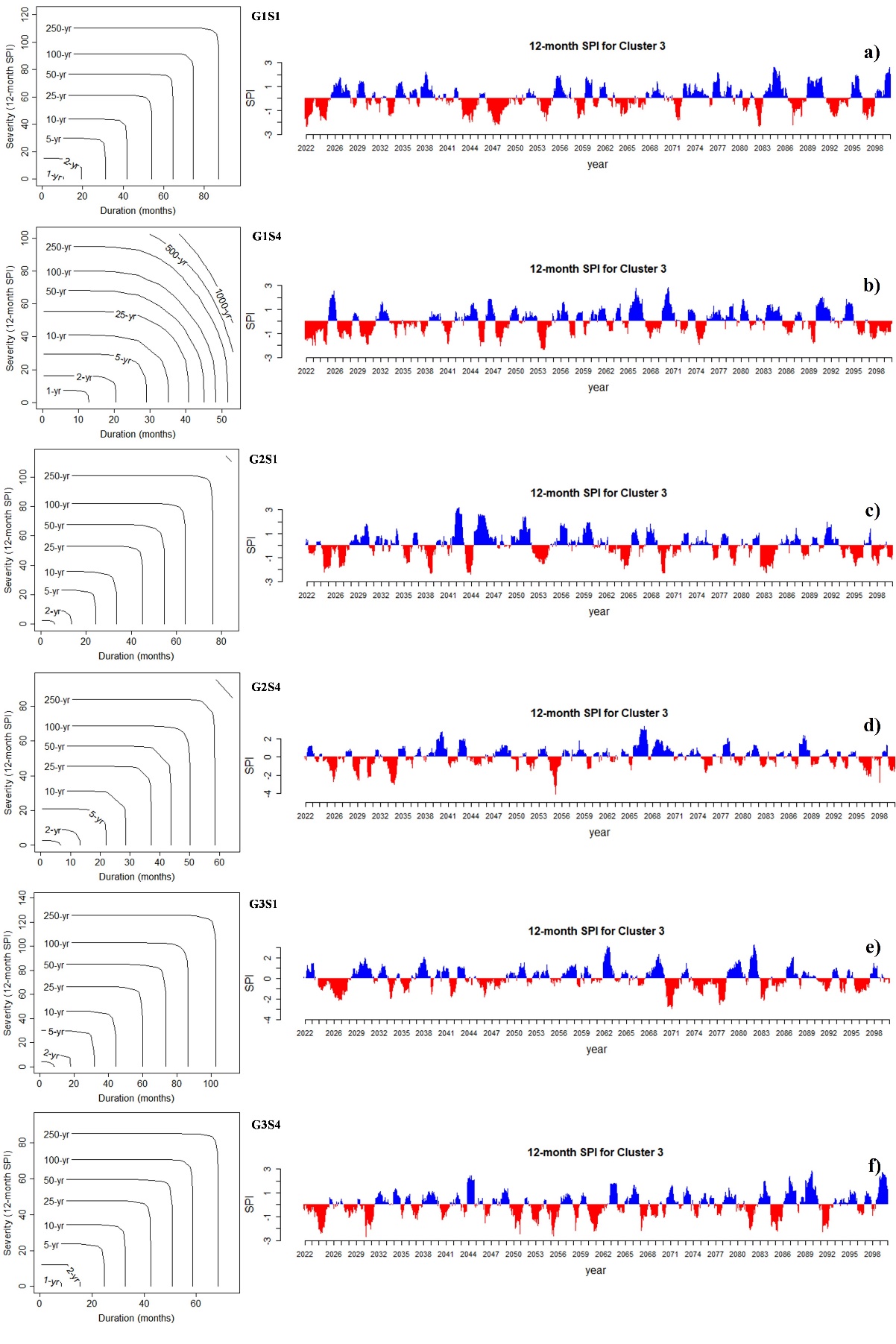


**Supplementary Figure 3** Joint drought returns periods (years) and meteorological drought index (SPI) on a 12-month scale in the future period (2022-2100) for cluster 3 in Iran. a) G1S1, b) G1S4, c) G2S1, d) G2S4, e) G3S1 and f) G3S4.


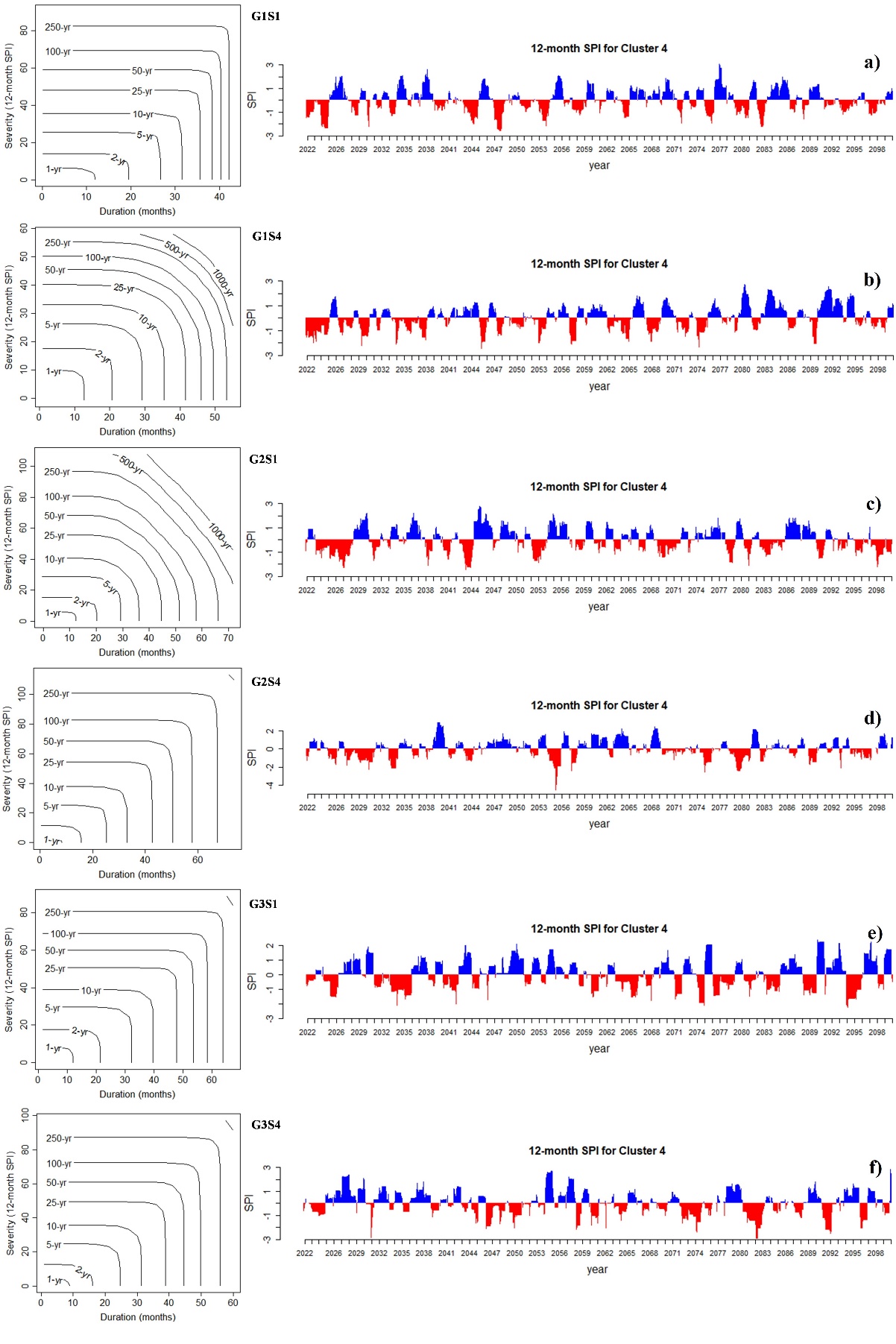


**Supplementary Figure 4** Joint drought returns periods (years) and meteorological drought index (SPI) on a 12-month scale in the future period (2022-2100) for cluster 4 in Iran. a) G1S1, b) G1S4, c) G2S1, d) G2S4, e) G3S1 and f) G3S4.


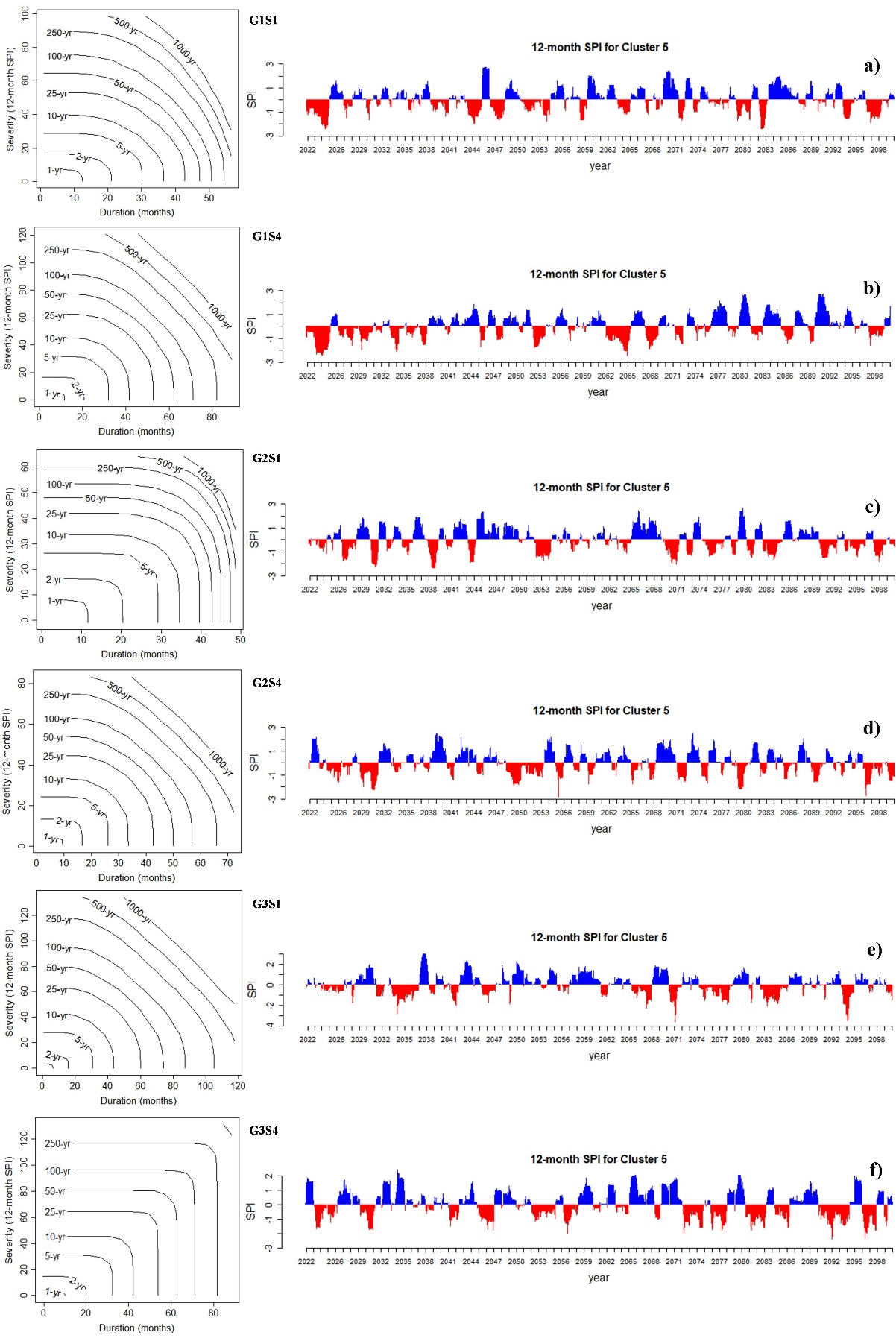


**Supplementary Figure 5** Joint drought returns periods (years) and meteorological drought index (SPI) on a 12-month scale in the future period (2022-2100) for cluster 5 in Iran. a) G1S1, b) G1S4, c) G2S1, d) G2S4, e) G3S1 and f) G3S4.


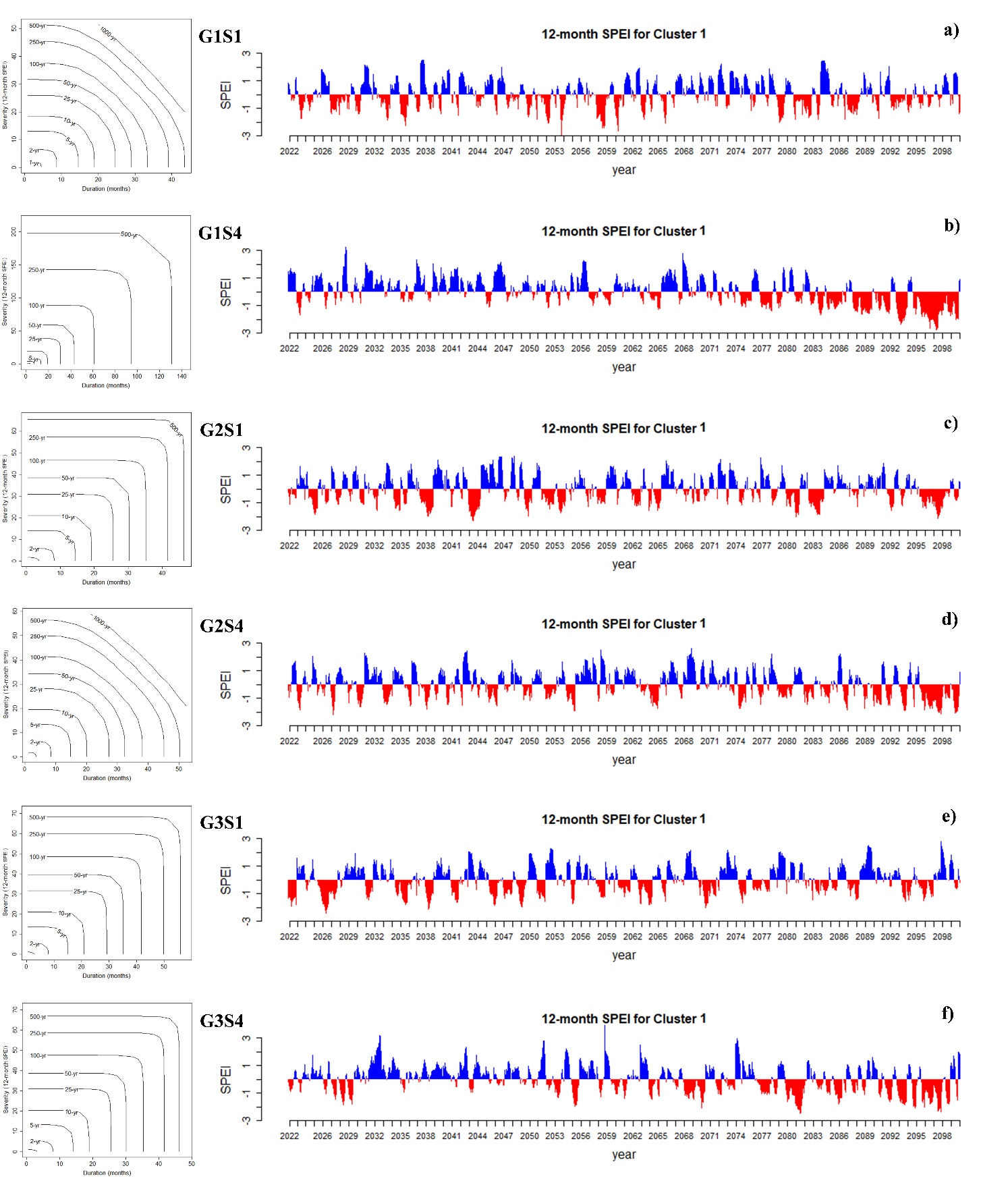


**Supplementary Figure 6** Joint drought returns periods (years) and Standardized Precipitation Evapotranspiration Index (SPEI) on a 12-month scale in the future period (2022-2100) for cluster 1 in Iran. a) G1S1, b) G1S4, c) G2S1, d) G2S4, e) G3S1 and f) G3S4.


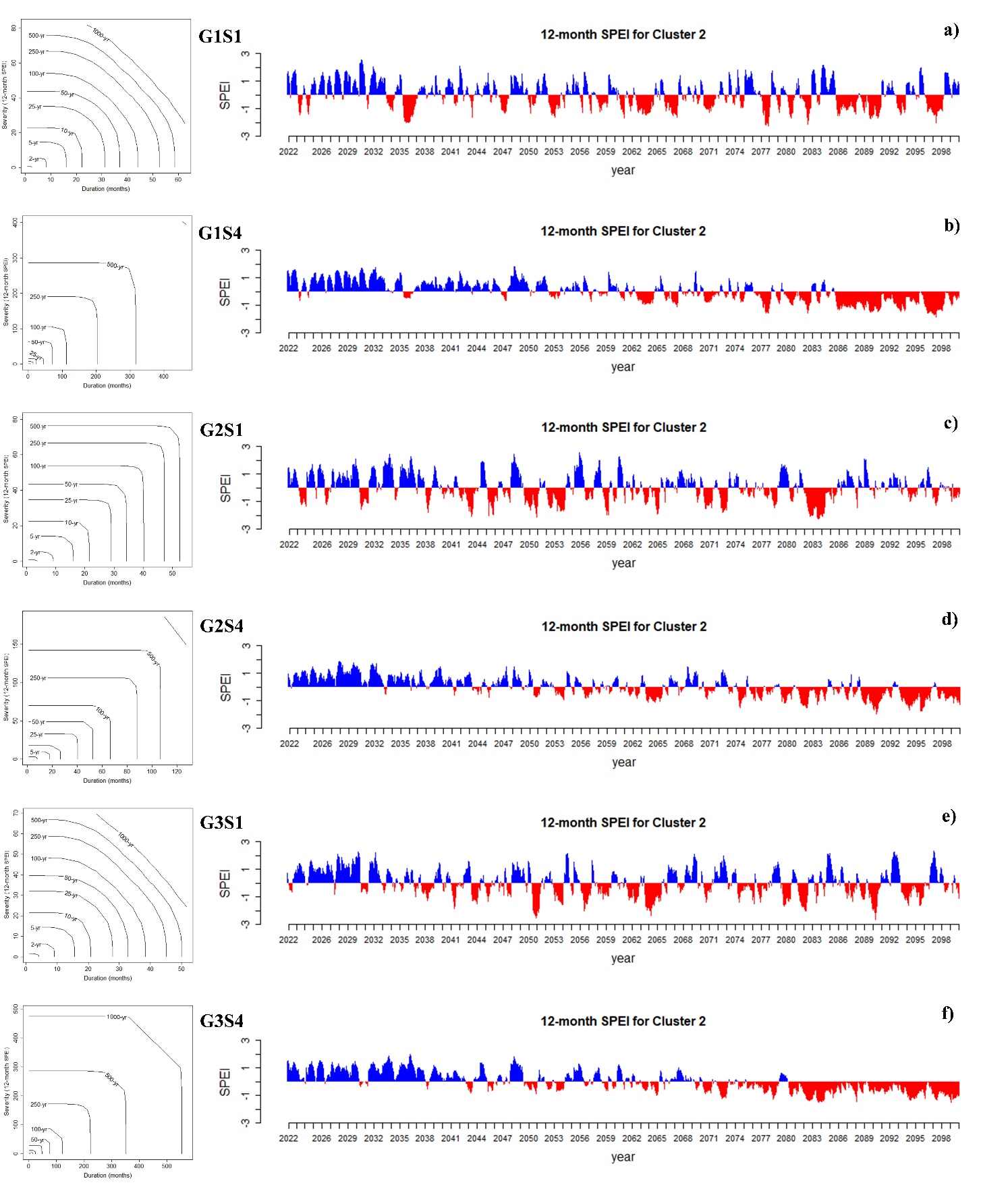


**Supplementary Figure 7** Joint drought returns periods (years) and Standardized Precipitation Evapotranspiration Index (SPEI) on a 12-month scale in the future period (2022-2100) for cluster 2 in Iran. a) G1S1, b) G1S4, c) G2S1, d) G2S4, e) G3S1 and f) G3S4.


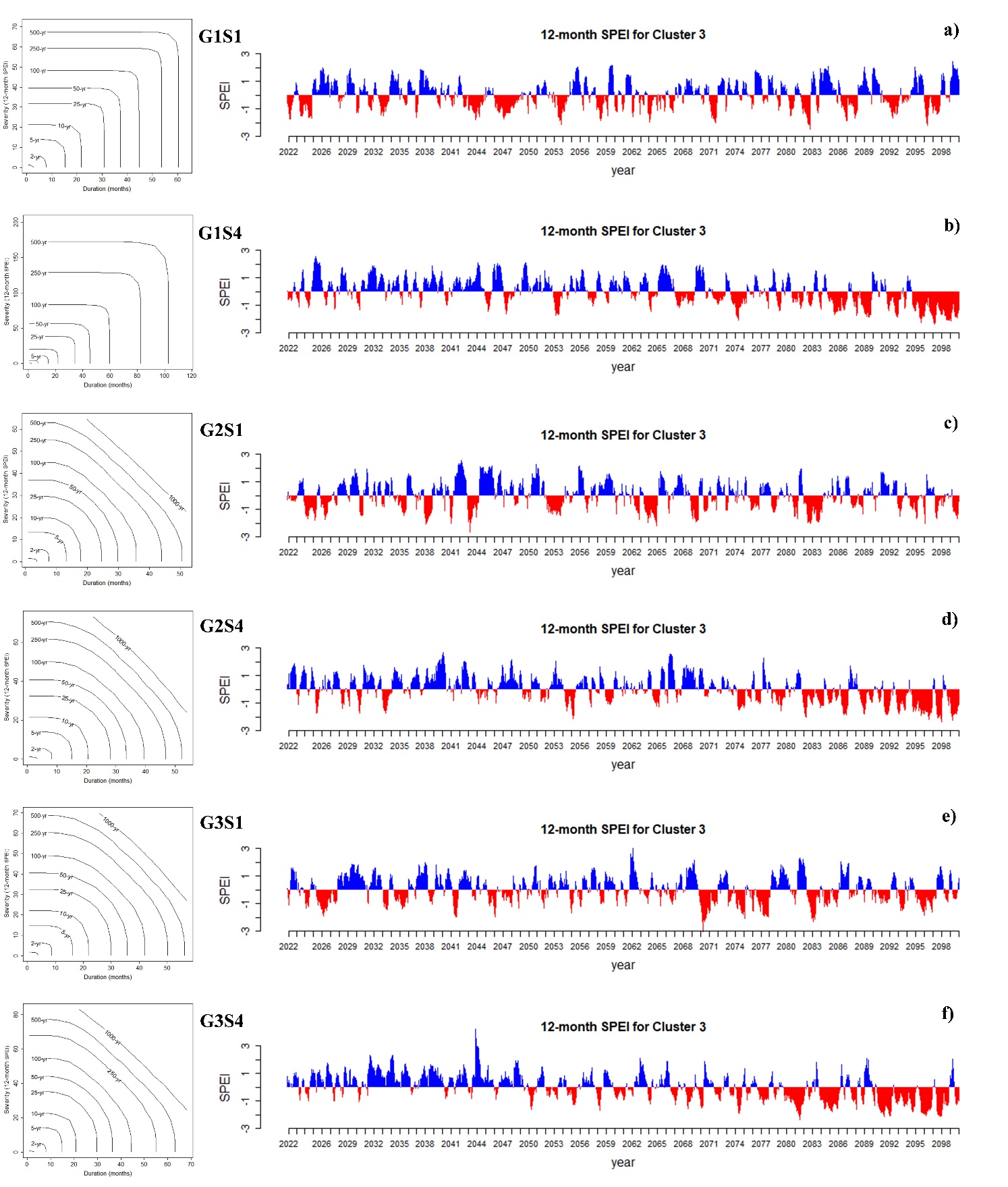


**Supplementary Figure 8** Joint drought returns periods (years) and Standardized Precipitation Evapotranspiration Index (SPEI) on a 12-month scale in the future period (2022-2100) for cluster 3 in Iran. a) G1S1, b) G1S4, c) G2S1, d) G2S4, e) G3S1 and f) G3S4.


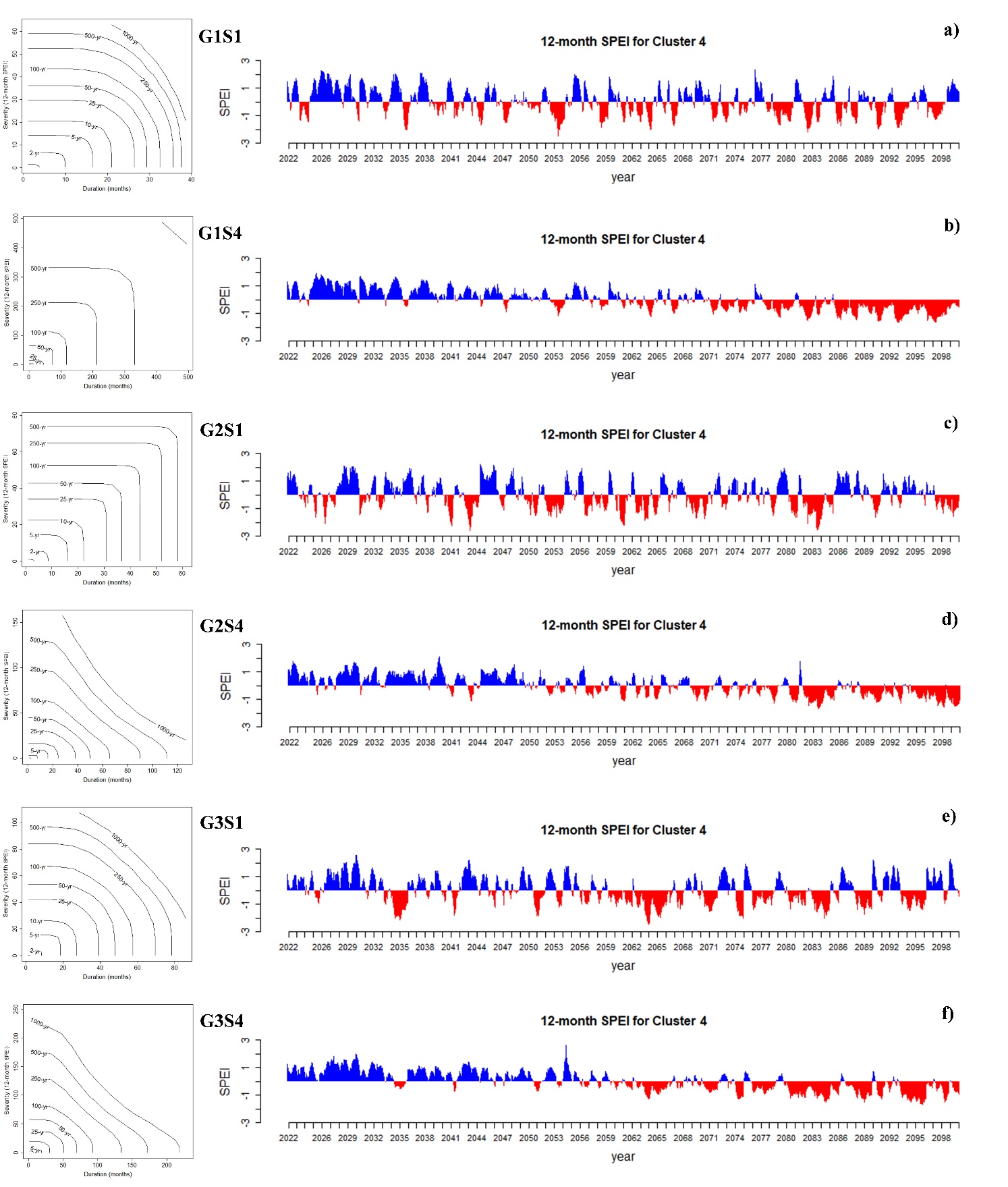


**Supplementary Figure 9** Joint drought returns periods (years) and Standardized Precipitation Evapotranspiration Index (SPEI) on a 12-month scale in the future period (2022-2100) for cluster 4 in Iran. a) G1S1, b) G1S4, c) G2S1, d) G2S4, e) G3S1 and f) G3S4.


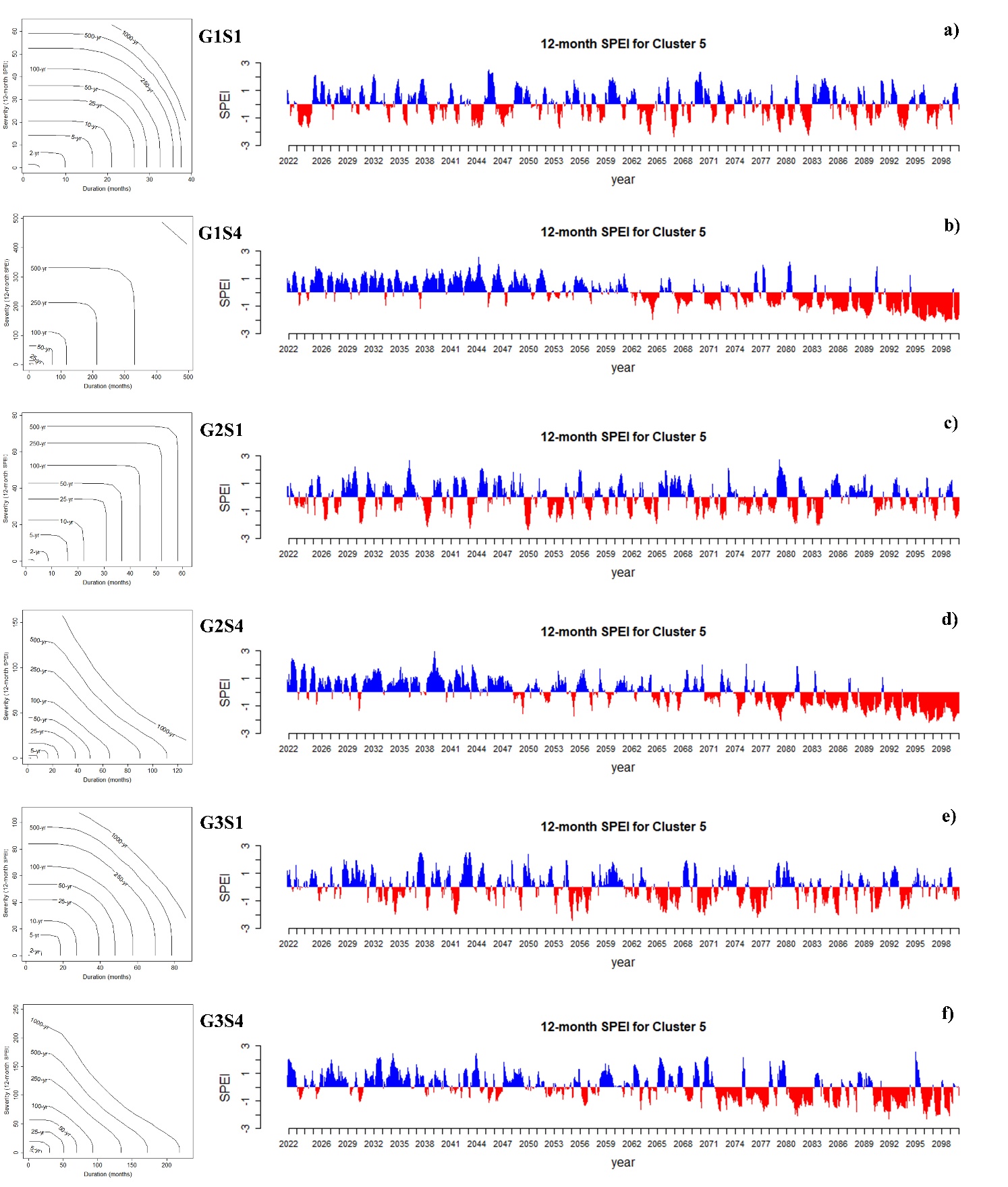


**Supplementary Figure 10** Joint drought returns periods (years) and Standardized Precipitation Evapotranspiration Index (SPEI) on a 12-month scale in the future period (2022-2100) for cluster 5 in Iran. a) G1S1, b) G1S4, c) G2S1, d) G2S4, e) G3S1 and f) G3S4.

**Supplementary Figure 11** Heat map of the performance of CMIP6 models in cluster 1.

**Supplementary Figure 12** Heat map of the performance of CMIP6 models in cluster 2.

**Supplementary Figure 13** Heat map of the performance of CMIP6 models in cluster 3.

**Supplementary Figure 14** Heat map of the performance of CMIP6 models in cluster 4.

Supplementary Figure 15 Heat map of the performance of CMIP6 models in cluster 5.
